# Evidence of protective role of Ultraviolet-B (UVB) radiation in reducing COVID-19 deaths

**DOI:** 10.1038/s41598-020-74825-z

**Published:** 2020-10-19

**Authors:** Rahul Kalippurayil Moozhipurath, Lennart Kraft, Bernd Skiera

**Affiliations:** Faculty of Economics and Business, Goethe University Frankfurt, Theodor-W.-Adorno-Platz 4, 60629 Frankfurt, Germany

**Keywords:** Biochemistry, Biological techniques, Biotechnology, Cell biology, Chemical biology, Computational biology and bioinformatics, Drug discovery, Immunology, Microbiology, Molecular biology, Physiology, Structural biology, Systems biology, Biomarkers, Cardiology, Diseases, Endocrinology, Health care, Health occupations, Medical research, Molecular medicine, Pathogenesis, Risk factors, Signs and symptoms

## Abstract

Prior studies indicate the protective role of Ultraviolet-B (UVB) radiation in human health, mediated by vitamin D synthesis. In this observational study, we empirically outline a negative association of UVB radiation as measured by ultraviolet index (UVI) with the number of COVID-19 deaths. We apply a fixed-effect log-linear regression model to a panel dataset of 152 countries over 108 days (n = 6524). We use the cumulative number of COVID-19 deaths and case-fatality rate (CFR) as the main dependent variables and isolate the UVI effect from potential confounding factors. After controlling for time-constant and time-varying factors, we find that a permanent unit increase in UVI is associated with a 1.2 percentage points decline in daily growth rates of cumulative COVID-19 deaths [p < 0.01] and a 1.0 percentage points decline in the CFR daily growth rate [p < 0.05]. These results represent a significant percentage reduction in terms of daily growth rates of cumulative COVID-19 deaths (− 12%) and CFR (− 38%). We find a significant negative association between UVI and COVID-19 deaths, indicating evidence of the protective role of UVB in mitigating COVID-19 deaths. If confirmed via clinical studies, then the possibility of mitigating COVID-19 deaths via sensible sunlight exposure or vitamin D intervention would be very attractive.

## Introduction

COVID-19 is causing significant economic, healthcare and social disruption globally. However, it is not yet known how to prevent or treat COVID-19. Prior studies indicate the protective role of Ultraviolet-B (UVB) radiation in human health. UVB radiation exposure is a major source of vitamin D, which increases immunity and reduces the likelihood of severe infections and mortality.

A recent COVID-19 study indicates abnormally high case-fatality-rate (CFR) of 33.7% among nursing home residents^1^, which is consistent with studies indicating higher prevalence of vitamin D deficiency among them because of their lower mobility^2,3^. Increasingly, studies establish a link between vitamin D deficiency and comorbidities such as cardiovascular disease^4^, hypertension^5^, obesity^2,6^, type 1, and type 2 diabetes^7^. This evidence is consistent with clinical studies in China and Italy that indicate comorbidities such as hypertension, diabetes and cardiovascular diseases could be important risk factors for critical COVID-19 cases^8–10^. Epidemiology of COVID-19 provides evidence that vitamin D might be helpful in reducing risk associated with COVID-19 deaths^11,12^. If such a link is true, then it will be cost-effective to mitigate COVID-19 via sensible exposure to sunlight or via vitamin D nutritional intervention. Yet, to the best of our knowledge, so far, no empirical study has used data across many countries to explore the association between UVB radiation as measured by ultraviolet index (UVI) and the number of deaths attributed to COVID-19 (COVID-19 deaths).

The aim of this study is therefore to examine the relation of UVB radiation, as measured by ultraviolet index (UVI), with the number of COVID-19-deaths. The results of our study demonstrate that a one-unit increase in UVI is associated with a 1.2 percentage points decline in daily growth rates of cumulative COVID-19 deaths. The robustness checks confirm the stability of our results because they show a similar effect of UVI on case fatality rate (effect size: − 0.010) and comparable results across a variety of different model specifications (effect size: − 0.006 to − 0.012).

A major threat to identifying the effect of UVB with the number of COVID-19 deaths is the presence of time trends, which could affect UVI as well as the number of COVID-19 deaths. For example, many countries affected by COVID-19 in spring 2020 are in the northern hemisphere leading to a natural phenomenon that UVI increases over time. In addition, growth rates of the cumulated COVID-19 deaths are decreasing over time. This negative correlation between UVI and the cumulated COVID-19 deaths due to time is the source of the identification problem. We address this problem through our statistical analysis in which we flexibly isolate UVI from linear or non-linear time trends which can be either similar across countries or even country-specific.

## Importance of UVB radiation for human health

Prior studies find that UVB radiation plays a protective role in human health because it reduces the severity of immune diseases^13^, reduces the risk of getting cancer—e.g., prostate cancer^14^ and dying from cancer^15,16^ and may reduce the prevalence of hypertension^17^.

Humans receive vitamin D either from their diet (natural food, fortified food or supplements) or from skin synthesis by solar UVB radiation exposure^18^. Vitamin D levels are also associated with dietary patterns. For example, vegetarians and vegans tend to have lower vitamin D levels than meat and fish eaters^19^. In general, skin synthesis is the major source of vitamin D^20,21^, as the dietary intake is usually insufficient^22^. Various studies consider that UVB exposure twice a week is sufficient to maintain vitamin D levels^22^ and that vitamin D once produced can be stored in body fat and can be utilized later^22^, indicating a lagged effect of UVB.

**Figure 1:**
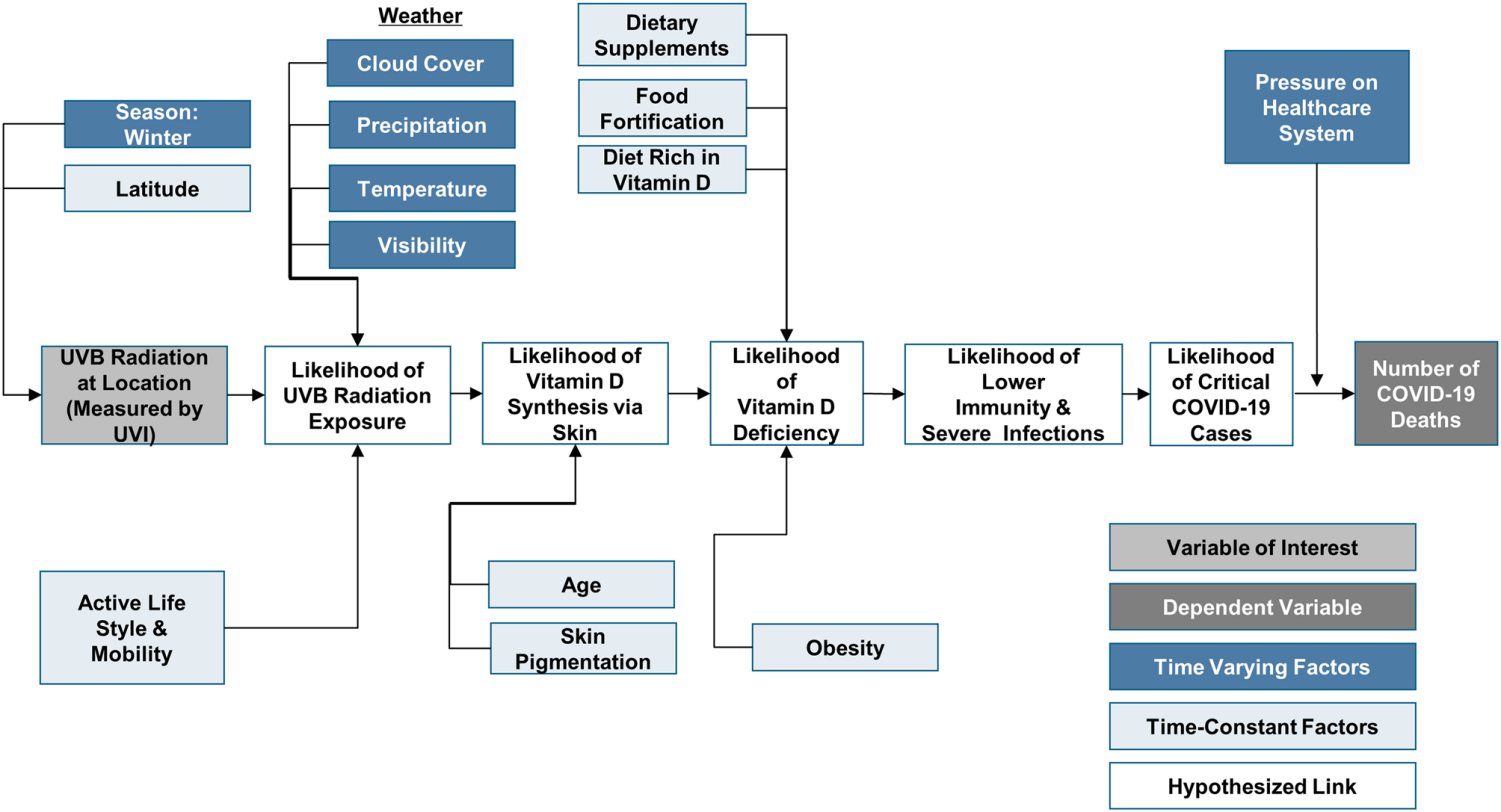
Explanation of protective role of Ultraviolet-B (UVB) radiation in COVID-19 deaths mediated by vitamin D synthesis and deficiency.

UVB radiation varies significantly across latitudes, seasons and time of the day. Specifically, during winter months in northern latitudes (e.g., above 35° latitude—Oklahoma, USA), the ozone absorbs most of the UVB^23^, leading to a reduced likelihood of UVB radiation exposure and thereby insufficient vitamin D synthesis as indicated in Fig. 1.

Studies indicate that the transmission dynamics of viruses are associated with weather factors^24^ such as humidity^25^, UV index^3^, temperature^26^ and precipitation^26,27^. Influenza, a viral disease which affects the respiratory tract, shows seasonality and tends to peak in winter^28^ coinciding with low temperature^26,29^, low humidity^30,31^ and low UV Index^26^. The influence of meteorological factors on seasonal influenza may be primarily due to their impact on viral survival and transmission^30,32^. Studies also indicate that the seasonality of influenza in higher latitude countries may be partly due to the seasonal variation of UVB radiation and the associated vitamin D skin synthesis^11,33,34^ . Grant et al.^11^ reviews the literature on influenza epidemiology and COVID-19 characteristics providing an early indication that vitamin D intervention may lower the risks associated with seasonal influenza and COVID-19^11^.

Early COVID-19 evidence indicates that significant outbreaks are associated with weather factors such as temperature and humidity^27,35^. Furthermore, weather factors such as cloud cover, precipitation, visibility and temperature influence the likelihood of exposure to UVB radiation and thereby vitamin D deficiency due to reduced skin synthesis. For example, clouds not only reduce the amount of UVB radiation but also the likelihood of UVB radiation exposure as people are more likely to undertake outdoor activities on less cloudy days. Therefore, we control for these time-varying confounding factors to isolate the protective role of UVB Radiation.

Lifestyle and mobility also influence the likelihood of UVB radiation exposure^3,36,37^. Similarly, the likelihood of vitamin D deficiency increases with age^22^, skin pigmentation^38^ and obesity due to reduced skin synthesis^39^. Specifically, people with darker skin pigment require higher UVB exposure compared to those with lighter pigmented skin to synthesize similar levels of vitamin D^38^.

Studies indicate that UV radiation may help in reducing the likelihood of transmission by inactivating viruses in transmission^40^. However, UVB radiation also plays another protective role via vitamin D skin synthesis. Since UVB radiation exposure is a major source of vitamin D, an increase in the likelihood of skin exposure to UVB radiation increases vitamin D synthesis, thereby reducing the likelihood of vitamin D deficiency. Therefore, different time-varying and time-constant factors influencing the UVB radiation variation and exposure also influence the likelihood of vitamin D synthesis and thereby deficiency.

Prior studies indicate that vitamin D deficiency increases the likelihood of weakened immune response^18,41,42^, infectious diseases in the upper respiratory tract^22,43,44^ and the severity as well as mortality in critically ill patients^45^. From a biological perspective, there are several reasons to hypothesize that vitamin D may reduce the risk of severe cases and deaths in COVID-19^11,46^. Vitamin D, through its active form, 1,25-dihydroxyvitamin D [1,25 (OH)_2_D], plays an essential role in the immunomodulation of both—innate and adaptive immune systems^11,46^. Vitamin D, via its active form, enhances innate immunity by the stimulation of antimicrobial peptides such as defensins and human cathelicidin, with anti-viral effects such as the ability to disrupt viral envelopes^11,46–48^. It also modulates the inflammatory response by suppressing the excessive expression of proinflammatory cytokines, thereby reducing the risk of cytokine storm^11,46^. Studies indicate that vitamin D, through its active form, 1,25 (OH)_2_D, may also play a protective role in modulating renin-angiotensin system (RAS), specifically in regulating the expression of ACE2 (angiotensin-converting enzyme-2)^46,49,50^. Overactivation of renin-angiotensin system (RAS) is increasingly associated with poor clinical outcomes in COVID-19^51,52^. Emerging epidemiological and clinical evidence related to COVID-19 also suggests that vitamin D deficiency is associated with an increased likelihood of COVID-19 incidence^53–55^, severity^56^ and mortality^57^, further providing evidence for the vitamin D mediated protective role of UVB radiation.

Therefore, we expect that an increased skin synthesis of vitamin D due to increased UVB radiation increases the likelihood of immunity and reduces the likelihood of severe infections, thereby reducing the critical COVID-19 cases. Thus, we anticipate that an increase in UVB radiation as measured by ultraviolet index (UVI) relates to a reduction of the number of COVID-19 deaths. Figure 1 summarizes these different factors that explain the potential protective role of UVB radiation in reducing COVID-19 deaths, mediated by vitamin D synthesis and deficiency.

## Methods

### Description of data

In order to identify the relation of UVB radiation and COVID-19 deaths, we constructed the dataset outlined in Table 1. We collected data covering 108 days from 22 January 2020 until 8 May 2020 across 183 countries of which 158 reported the number of COVID-19 deaths prior to 8 May 2020 and of which 152 reported more than 20 COVID-19 infections prior to 8 May 2020. We focus on those 152 countries to ensure that the results are not biased by countries that are at a very early stage of the COVID-19 outbreak, which would limit data points with respect to COVID-19 deaths. In addition, we drop the first 20 daily observations of every country after that country reported the first COVID-19 infection to further ensure that results are not biased by the observations at the very early stage of the COVID-19 outbreak.

**Table 1:** Summary of dataset.

Number of countries in the world	195
Number of countries in our dataset	183
… > 0 cumulated number of COVID-19 deaths before 8 May 2020	158
… > 20 cumulated number of COVID-19 infections before 8 May 2020	152
Covered time-period	22 January 2020–8 May 2020 (108 days)
Granularity of data	Daily
COVID-19 data source (John Hopkins University)	https://github.com/ CSSEGIS and Data/COVID-19
Latitude and longitude data source for each country that is used to match weather data (John Hopkins University)	https://github.com/ CSSEGIS and Data/COVID-19
Weather data source	https://darksky.net/

The corresponding country level data consist of the cumulative daily number of COVID-19 deaths and infections. They also consist of the daily ultraviolet index (UVI), which is closely connected to the daily UVB radiation, and a set of control variables such as daily weather parameters that include precipitation index, cloud index, ozone level, visibility level, humidity level, minimum and maximum temperature. We source COVID-19 data from John Hopkins University^58^ and the weather data from darksky.net based on the latitude and longitude information of countries that are provided by John Hopkins University.

We present descriptive statistics of the dataset in Table 2. As of 8th of May, 2020, the cumulative COVID-19 deaths of these 152 countries were on average 1,800 and the growth rate of COVID-19 deaths across all countries on 8 May was on average 2.6% as compared to the average growth rate of COVID-19 deaths across all countries and time which was 10%. The cumulative COVID-19 infections per country were on average 26,000. The case-fatality-rate (CFR), as measured by the cumulative COVID-19 deaths divided by the cumulative COVID-19 infections per country, was on average 4.3% on 8 May. The growth rate of CFR on 8 May was on average -1.1% as compared to the growth rate of CFR across countries and time which was 2.6%. We use cumulative COVID-19 deaths as the main dependent variable to test our hypothesis linking UVB radiation to COVID-19 deaths and use the CFR to test the consistency of our results. On average, the first reported COVID-19 infection in each country happened 68 days before 8 May 2020. UVI is on average 6.8 representing a moderate to high risk of harm from unprotected sun exposure.

**Table 2:** Descriptive statistics of data set.

Variable	Number of countries	Number of observations	Mean	Std. dev	Min	Max
Cumulated COVID-19 deaths on 8 May	152	152	1,800	7,800	1	77,000
Growth rate of cumulative COVID-19 deaths on 8 May	152	152	0.026	0.051	0	0.4
Daily growth rate of cumulative COVID-19 deaths	152	6589	0.10	0.26	− 1	9
Cumulated COVID-19 infections on 8 May	152	152	26,000	111,000	23	1,284,000
CFR on 8 May	152	152	0.043	0.037	0.001	0.21
Growth rate of CFR on 8 May	152	152	− 0.011	0.057	− 0.42	0.24
Daily growth rate of CFR	152	6589	0.026	0.20	− 1	5.9
Time-passed by from first reported infection until 8 May	152	152	68	18	29	108
Daily ultraviolet index (UVI)	152	7471	6.8	3.1	0	14
Daily precipitation index	152	7471	0.29	0.31	0	1
Daily cloud index	152	7471	0.50	0.30	0	1
Daily ozone level	152	7471	308	47	236	473
Daily visibility level	152	7471	15	2.2	0.12	16
Daily humidity level	152	7471	0.63	0.20	0.04	1
Minimum temperature per day within a country	152	7471	12	10	− 23	31
Maximum temperature per day within a country	152	7471	23	10	− 16	46

### Illustration of ultraviolet index (UVI) and COVID-19 deaths

**Figure 2:**
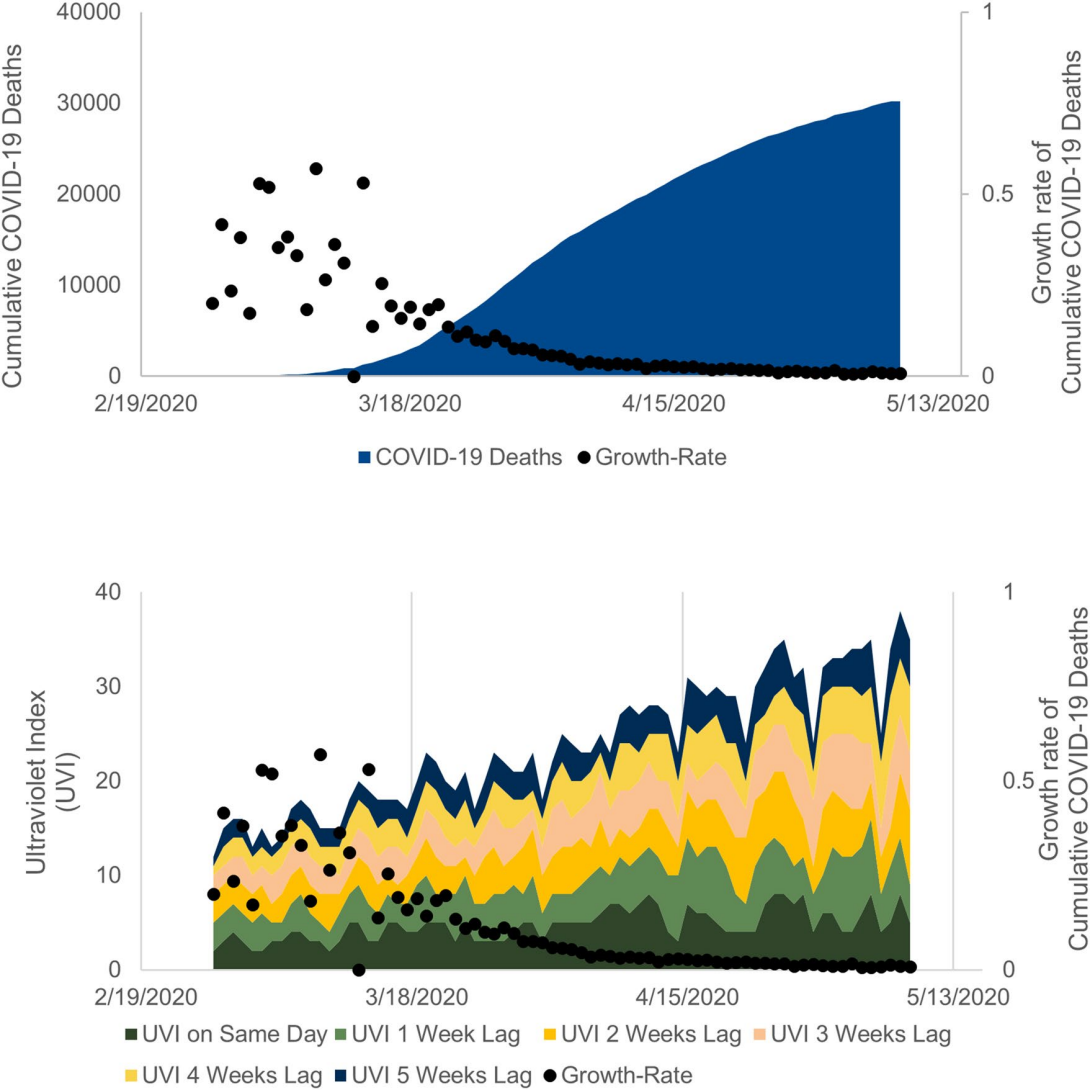
Cumulative number and growth rates of COVID-19 deaths and Ultraviolet index (UVI) for Italy.

Figure 2 shows the cumulative COVID-19 deaths and the associated daily growth rates for Italy from 26 February 2020 until 8 May 2020. As time progresses, the cumulative COVID-19 deaths increase but at a slower rate. Initially, the growth rate is high at 42% (growth rate from 26 to 27 February) and it gradually slows to 0.8% (growth rate from 7 to 8 May).

Figure 2 also shows the daily growth rates and daily UVI of Italy as well as the UVI values lagged by one, two, three, four and five weeks respectively. It is important to consider the lagged effect of UVI because synthesized vitamin D is cumulative and can be used later because it is stored in body fat^22^. Therefore, it seems more plausible that an increase of UVI today will continue to support an individual’s immunity later i.e., in two or more weeks. Furthermore, the likelihood of skin synthesis is low in severely infected people, while they are hospitalized, indicating the importance of lagged UVI values.

It is evident that the growth rates slow down over the observation period, as counter-measures imposed by governments take effect, which results in lower infection rates and lower mortality rates. At the same time, the UVI in the northern hemisphere countries is increasing due to seasonal changes from January to May. In order to approximate the association of UVI with cumulative COVID-19 deaths, we need to isolate it from the underlying time-trends, which are potentially affecting both UVI as well as the growth rates of cumulative COVID-19 deaths.

## Results

We estimate the effect of UVI on the cumulative COVID-19 deaths by using log-linear fixed-effects regression. The effect of UVI is isolated from time-constant country-specific factors (see Fig. 1) by using a within-transformation of the transformed structural model as outlined in Eq. (1) in Supplementary Appendix 1. Further, we use the partialling-out property to isolate the effect of UVI from all linear as well as some non-linear effects of time-varying factors such as weather and time, which may confound the results. Our statistical analysis is outlined in detail in *Description of Methodology* section in Supplementary Appendix 1.

The key finding is the significant negative long-run association of UVI on cumulative COVID-19 deaths. As we outline in the *Identification of UVI Effect* section in Supplementary Appendix 1, the estimate is likely to identify an upper bound of the relation, indicating that the association could be even stronger. Our results presented in Table 3 suggest that a permanent unit increase of UVI is associated with a decline of 1.2 percentage points in daily growth rates of cumulative COVID-19 deaths [p < 0.01]. Relative to the average daily growth rate of cumulative COVID-19 deaths (10%), this decline translates into a significant percentage change of − 12% (= − 1.2%/10%). We further find that a permanent unit increase of UVI is associated with a decline of 1.0 percentage points in the daily CFR growth rate [p < 0.05]. Compared with the average daily growth rate of CFR (2.6%), this decline translates into a significant percentage change of − 38% (= − 1.0%/2.6%).

**Table 3:** Effect of UVI on cumulative COVID-19 deaths.

	Model 1	Model 2
	COVID-19 deaths	CFR
**Dependent variable**		
L0.UVI	− 0.002 (− 1.53)	− 0.001 (− 0.41)
L1.UVI	0.000 (0.02)	− 0.001 (− 0.37)
L2.UVI	− 0.002 (− 1.03)	− 0.004* (− 2.18)
L3.UVI	− 0.002 (− 1.29)	− 0.002 (− 1.49)
L4.UVI	− 0.003* (− 2.03)	− 0.003 (− 1.53)
L5.UVI	− 0.002 (− 1.23)	0.000 (0.08)
Long− run coefficient	− 0.012** (F: 8.33)	− 0.010* (F: 6.23)
**Control variables**		
Time trend of growth rate	Linear	Linear
Country fixed-effects	Yes	Yes
Precipitation index	Yes	Yes
Cloud index	Yes	Yes
Ozone level	Yes	Yes
Visibility level	Yes	Yes
Humidity level	Yes	Yes
Temperature (min and max)	Yes	Yes
Number of estimates	49 (+ 152 FE)	49 (+ 152 FE)
Number of observations	6524	6524
Number of countries	152	152
R-squared within	13.74%	1.80%

t-statistics based on robust standard errors in parentheses. F-statistic for long-run coefficient in parentheses. L0.UVI stands for the effect of UVI at time t on the cumulated number of COVID-19 deaths at the same time, whereas L1.UVI, L2.UVI, L3.UVI, L4.UVI and L5.UVI stand for the effect of UVI lagged by 1, 2, or 3, 4 and 5 weeks respectively. FE stands for country fixed-effects.

^+^p < 0.10, *p < 0.05, **p < 0.01.

The results indicate no significant association from an increase of UVI on cumulative COVID-19 deaths on the same day or a week ahead. This insignificant finding is consistent with the fact that severely infected people are more likely to be hospitalized and therefore less likely to be exposed to UVB radiation during their hospital stay. We further recognize that UVB radiation may not make a real difference if someone is already severely infected and developed severe complications. The results also show that UVI has a stronger relation to COVID-19 deaths than CFR. We anticipate that the weaker association with CFR is plausible as UVI helps in vitamin D synthesis, making the infection less severe due to increased immunity, thereby prompting fewer people to take the test.

The results of the robustness checks presented in Table S2 and Table S3 (*Robustness Checks* section in Supplementary Appendix 1) suggest that the relation of UVI on cumulative COVID-19 deaths is consistent (between − 0.006 and − 0.012) across different model specifications which isolate the association of UVI from underlying time trends in flexible ways. In fact, the most flexible model—Model 8 of Table S3 (*Robustness Checks* section in Supplementary Appendix 1)—reveals substantial and significant evidence of the UVI relation with cumulative COVID-19 deaths (− 0.008, p < 0.05). The results of the robustness checks in Table S9 and Table S10 (*Robustness Checks* section in Supplementary Appendix 1) suggest that the association of UVI with cumulative COVID-19 deaths is stable even after considering governmental measures such as the lockdown (between − 0.007 and − 0.012).

**Figure 3:**
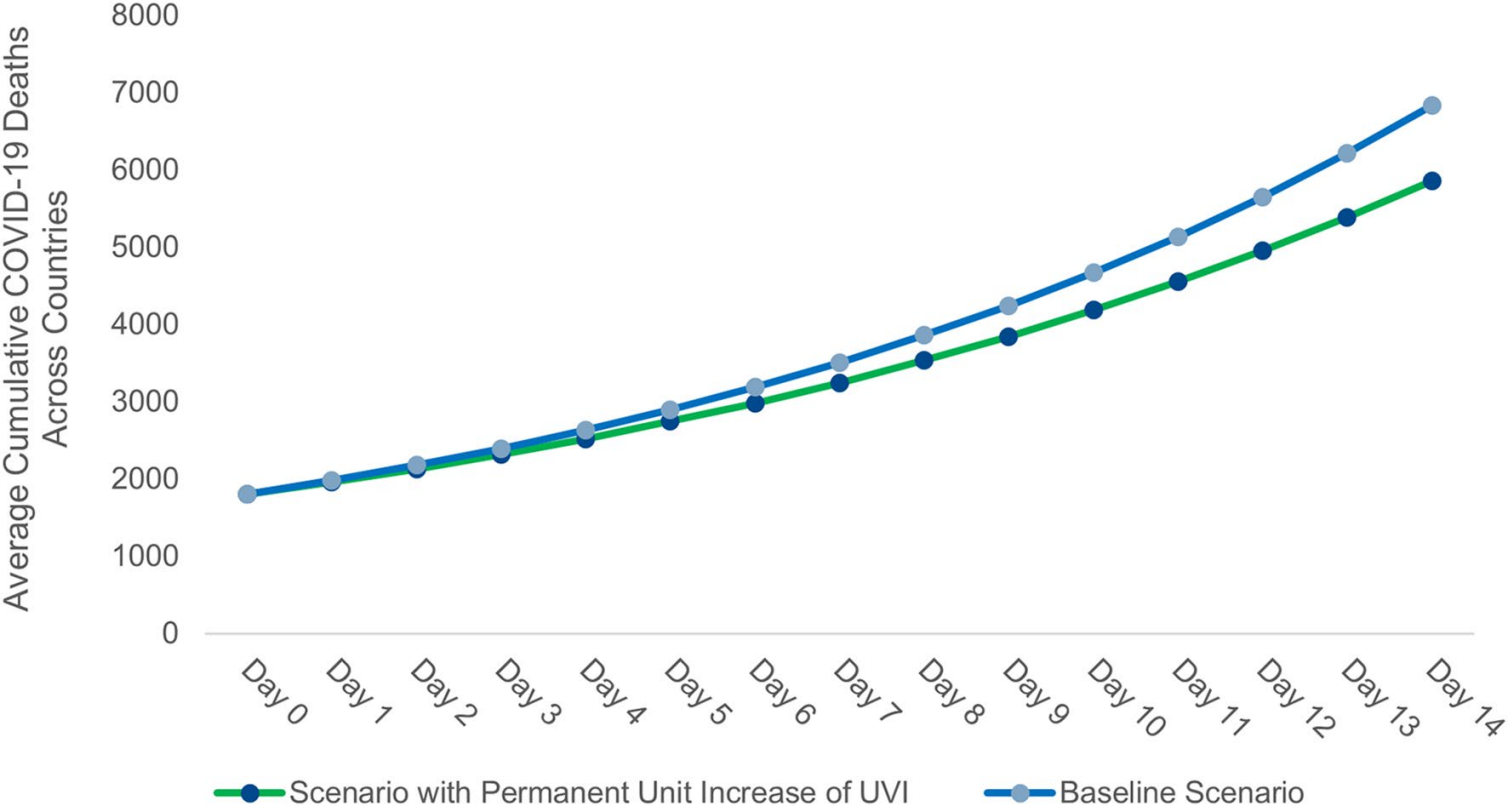
Long-run effects of a permanent unit increase of Ultraviolet index (UVI) on average cumulative COVID-19 deaths across countries.

Figure 3 outlines that the decline of 1.2 percentage points in daily growth rates of cumulative COVID-19 deaths has significant long-run effects on the cumulative COVID-19 deaths. In order to simulate the long-run effects, we take the average number of cumulative COVID-19 deaths across all 152 countries as of May 8, 2020, i.e., 1800 as cumulative COVID-19 deaths at day 0. Figure 3 also outlines a scenario with a permanent unit increase of UVI over the baseline scenario of average UVI of 6.8 across countries is associated with 1,000 or 14% fewer deaths in 14 days.

## Discussion

In this study, we find evidence of the protective role of UVB radiation in reducing COVID-19 deaths. Specifically, we find that a permanent unit increase in Ultraviolet index (UVI) is associated with a 1.2 percentage points decline in daily growth rates of COVID-19 deaths [p < 0.01] as well as a 1.0 percentage points decline in the daily growth rates of CFR [p < 0.05]. These results translate into a significant percentage reduction in terms of the daily growth rates of cumulative COVID-19 deaths (− 12%) and CFR (− 38%). Our results are consistent across different model specifications.

We control for all time-constant as well as various time-varying factors such as weather which may also have an effect on the transmission of the virus. We acknowledge that we may not be able to isolate the association of UVI with cumulative COVID-19 deaths from all other time-varying confounding factors. Still, we anticipate that an increased likelihood of immunity and a reduced likelihood of infections mediated by an increased likelihood of vitamin D synthesis may plausibly explain this finding. We also acknowledge that we may not be able to rule out the possibility of mediation by the reduced likelihood of transmission due to inactivation of the virus by UVB as well as via other UVB induced mediators—such as cis-urocanic acid, nitric oxide^13,40,59^.

As of 24 September 2020, some of the latest trends in daily deaths in selected northern and southern hemisphere countries across diverse latitude, hemisphere and demographics are in line with our findings^58^. Firstly, four of the worst affected European countries in terms of total deaths –UK, Italy, France and Spain, all with an older demographics—experienced their peak in daily deaths during late winter and early spring in the northern hemisphere^58^. The daily number of new infections in these countries increases again in late summer and early autumn, but not the daily deaths^58^. That relation between number of new infections and death was different in late winter and early spring^58^. Secondly, countries in the southern hemisphere like South Africa, Australia, Brazil and Chile show a declining trend after experiencing their daily deaths’ peaks in winter^58^. An exception is Argentina, where the daily deaths are still increasing. Thirdly, India, a sub-tropical northern hemisphere country with younger demographic—is still experiencing an increasing number of daily deaths coinciding with the monsoon season^58^.

Recent clinical studies also indicate an association between vitamin D deficiency and COVID-19 incidence^53–55^, severity^56^ and mortality^57^ that are in line with the results of our study. However, we acknowledge that our results only provide partial evidence of the protective role of UV radiation and, therefore, should not serve as a substitute for clinical studies^60^. Further clinical studies—observational or randomized controlled trials—are required to establish the casual relationship of vitamin D deficiency and COVID-19 deaths, potentially leading to a cost-effective policy intervention for the prevention or as a therapy for COVID-19. The possibility of mitigating COVID-19 via sensible exposure to sunlight or via vitamin D intervention seem to be very attractive from a policy maker’s perspective because of their low cost and side effects.

While sensible exposure to sunlight helps in synthesizing vitamin D, disproportionate exposure may also increase the risk of adverse health effects^22^. Solar UV radiation in general is associated with health risks such as actinic keratosis^61^, degenerative aging^62^ and wrinkles^62^. Excessive exposure to solar UV radiation can also cause sunburn^22^ and damage DNA in skin cells^62^. Studies indicate that disproportionate exposure to solar UV radiation is also associated with common skin cancer types such as melanoma (primarily Ultraviolet-A (UVA)), basal cell carcinoma (UVA and UVB) and squamous cell carcinoma (UVB)^63^. Therefore, disproportionate solar exposure needs to be avoided to mitigate any adverse health effects.

Various countries are implementing lockdown as a preventive measure to mitigate COVID-19 impact on healthcare system. Unfortunately, confinement at home also leads to limited UVB exposure and, thus, possibly increasing the risk of COVID-19 deaths. Countries could create awareness among the population regarding the importance of sensible exposure to sunlight, whilst continuing other measures such as social distancing as well as cautioning against disproportionate exposure. If confirmed via additional clinical studies, then countries could adopt a cost-effective vitamin D intervention program—especially among vulnerable populations with increased risk of vitamin D deficiency, e.g., elderly populations living in nursing homes, people with high body mass index, dark skinned people residing in higher latitudes, people with indoor lifestyle, or vegetarians.

## Limitations

In addition to time-varying weather factors, time-constant and time-varying human factors can affect COVID-19 deaths. Such time-constant human factors include the age distribution of the population, location, medical treatment system, chronic disease rate, dietary pattern, co-morbidities and proportion of people in care homes in a country because those factors hardly vary during our observation period. Time-varying human factors such as travel patterns, pollution, testing capacity and governmental measures such as lockdowns, wearing masks and social distancing may also affect COVID-19 deaths. Our methodology controls for all country-specific time-constant confounding factors as well as some of the time-varying confounding factors such as air pollution and the implementation of governmental measures, but has the following limitations.

Firstly, our method might be limited in capturing some of the time-varying factors. For example, time-varying factors such as changes in people’s behaviours likely associate with the change of seasons (and, thus, UVB variation) and COVID-19 deaths. Such time-varying behaviours include varying travel pattern of infected people, intake of more nutritious food and higher dietary supplement consumption due to the pandemic. Although we control for governmental measures, we do not have data on whether people consistently adhered to these governmental measures. We also do not have data on time-varying testing capacities of different countries. Secondly, our study does not use data on the level of vitamin D among the population in our countries that prevents the analysis of the relationship between COVID-19 deaths and vitamin D levels. Finally, our study cannot explain the disproportionate impact of COVID-19 among the elderly living in care homes, especially in many European countries^64–66^. We anticipate that solar UVB exposure is less likely to influence these deaths due to their age as well as their limited likelihood of exposure.

## Supplementary information

Supplementary file 1

## Data Availability

The data used in the study are from publicly available sources. Data regarding COVID-19 are obtained on 9th May 2020 from *COVID-19 Data Repository* by the *Center for Systems Science and Engineering (CSSE)* at *Johns Hopkins University* and can be accessed at https://github.com/CSSEGISandData/COVID-19. Data regarding weather is obtained from *Dark Sky* on the 9th May, 2020 and can be accessed at https://darksky.net/. We will make specific data set used in this study available for any future research. Interested researchers can contact one of the authors via email to get access to the data.
