# Supplementary material for: Evidence of protective role of Ultraviolet-B (UVB) radiation in reducing COVID-19 deaths
Source: Sci Rep. 2020 Oct 19;10:17705. doi: 10.1038/s41598-020-74825-z (PMC7572372; doi:10.1038/s41598-020-74825-z)
Supplement: Supplementary file 1 [file 41598_2020_74825_MOESM1_ESM.pdf]

Evidence of Protective Role of Ultraviolet-B (UVB) Radiation in Reducing COVID-19 Deaths

Supplementary Appendix

Rahul Kalippurayil Moozhipurath*[1], Lennart Kraft[1], Bernd Skiera[1]

(Faculty of Economics and Business, Goethe University Frankfurt[1])

Rahul Kalippurayil Moozhipurath, PhD Student; Lennart Kraft, PhD Student; Bernd Skiera, Chaired Professor of Electronic Commerce and Recipient of an ERC Advanced Grant; Faculty of Economics and Business, Goethe University Frankfurt, Theodor-W.-Adorno-Platz 4, 60629 Frankfurt, Germany; email: rahulkm85@gmail.com, Phone: +49-152-1301-0589; email: lennart.kraft@wiwi.uni-frankfurt.de; Phone +49-69-798-34769; email: skiera@wiwi.uni-frankfurt.de, Phone +49-69-798-34649

Table of Contents

1 Description of Methodology 3
2 Derivation of Short- and Long-Run Effects 6
3 Identification of Effect of Ultraviolet Index (UVI) 6
4 Model Selection to Identify the Effect of Ultraviolet Index (UVI) on the Cumulative COVID-19 Deaths 9
5 Robustness Checks 9
6 Derivation of Equation (2) 12
7 Identification of Daily Growth Rate γ 13
8 Extended Regression Results (Fixed Effects Analysis) 14
9 Supplementary Tables 14
10 References 26

1 Description of Methodology

We apply a fixed-effect log-linear regression model to estimate the effect of UVI on the number of COVID-19 deaths that builds upon Fig. 1 in *Manuscript*. A log-linear model increases the comparability of the growth rates of COVID-19 deaths across countries because it considers percentage rather than absolute changes over time, and percentage growth rates are more comparable across countries than absolute ones. The model isolates the effect of UVI from country-specific time-constant factors. These time-constant factors consist of the countries' location (measured by its latitude and longitude) and diet-related effects such as dietary supplements, food fortification and diets which are rich in vitamin D. These time-constant factors also include the composition of vegans and vegetarians in the population, who tend to have lower vitamin D levels than meat and fish eaters. They also consist of other population parameters such as how active their people's lifestyle and mobility are and the composition of the population with respect to their age, skin pigmentation, obesity rates, underlying diseases, co-morbidities or treatments. Furthermore, they consist of the economic and social situation of a population in a country which remains to be fairly stable over our observation period.

Furthermore, the model allows to control for an increasing pressure on the healthcare system over time measured by the time passed by since the first reported case of COVID-19 in the specific country. Importantly, this factor would partial out any linear change of growth rates over time that is similar across countries. Therefore, the model isolates the effect of UVI from an exponential-shaped curve which is often observed in the cumulative COVID-19 deaths over time or in the growth rates of Fig. 2 in *Manuscript*. The model also isolates the effect of UVI from factors which can influence UVI or individuals' absorption of UVB such as precipitation index, cloud index, ozone level, visibility level, humidity level, and temperature.

Time-constant behaviours of individuals are captured by our country-fixed effects because these fixed effects capture time-constant reoccurring habits or mobility of individuals, which affect the likelihood of exposure to UVB radiation (e.g., walking to work, outdoor exercises, outdoor activities related to work.) Yet, country-fixed effects do not capture time-varying factors, in particular weather that influence the behaviour of individuals and thus the likelihood of UVB radiation exposure.

Because an increase of UVB today plausibly affects individuals several weeks later we include in our model five weekly lags of UVI and of the control variables. Additional lags did not change the results substantively. Thus, we use the following model of equation (1) to explain the number of COVID-19 deaths:

$$D_{i,t} = D_{i,t-1} \times e^{\gamma + \sum_{j=0}^{5}[UVI_{i,t-j}\beta_{UVI,j} + C_{i,t-j}\beta_{C,j}] + FI_{i,t}\beta_{FI} + u_i + \epsilon_{i,t}} \quad (1)$$

$D_{i,t}$ represents the cumulative COVID-19 deaths in country i at time point t (in days) and it is related to the explanatory factors via an exponential growth model on the right-hand side of the equation. The exponential growth model flexibly allows for different shapes of the cumulative COVID-19 deaths. These shapes cover the one described in Fig. 2 of the *Manuscript* or often observed S-shaped curves which appear at later stages of COVID-19 outbreaks, depending on how flexible time is allowed to enter the exponential growth model. The exponential growth model consists of six explanatory parts.

1) γ represents the daily growth rate of COVID-19 deaths from $D_{i,t-1}$ to $D_{i,t}$ that is independent of the factors presented in Fig. 1 in *Manuscript.* γ covers virus-specific attributes like its basic reproductive rate R_0 combined with its lethality.
2) $UVI_{i,t-j}$ represents the UVI for a country i at day t lagged by j weeks. $\beta_{UVI,j}$ reflects the effect of UVI lagged by j weeks.
3) $C_{i,t-j}$ stands for the set of control variables. This set consists of precipitation index, cloud index, ozone level, visibility level, humidity level, as well as minimum and

maximum temperature for a country i at day t lagged by j weeks. The vector $\beta_{C,j}$ identifies the effect of these control variables lagged by j weeks.

4) $FI_{i,t}$ stands for the time passed by since the first reported COVID-19 infection for a country i at day t and β_{FI} identifies the associated effect.

5) u_i represents time-constant country-specific factors influencing the growth rate of cumulative COVID-19 deaths (e.g., diet related effects, population parameters about their activities and demographic composition).

6) $\epsilon_{i,t}$ consists of all the remaining factors that are not identified but also have an effect on the cumulative COVID-19 deaths (i.e., all non-linear differences of growth rates with respect to time and country-specific linear differences of growth rates with respect to time). They could be caused by a decreasing number of people who could potentially become infected or contagious, lockdowns in a country , mutation of the virus in a country over time, systematic false-reports of the dependent variable.

An appropriate transformation (see Section 6 for the Derivation of Equation (2)) results in the estimable equation (2).

$$\Delta \ln\left(\ddot{D}_{i,t}\right) = \sum_{j=0}^{5} \left[\ddot{UVI}_{i,t-j}\beta_{UVI,j} + \ddot{C}_{i,t-j}\beta_{C,j}\right] + \ddot{FI}_{i,t}\beta_{FI} + \ddot{\epsilon}_{i,t} \qquad (2)$$

γ and u_i do not appear in the equation anymore and a linear regression can identify all other coefficients. Equation (2) also shows why we can only use those observations where cumulative COVID-19 deaths are larger than zero. For example, the first COVID-19 death of Italy was reported on 02/21/2020. Therefore, we can only include observations of Italy starting from 02/22/2020. This condition explains the difference between the 7,471 observations of 152 countries in Table 2 and the 6,524 observations of 152 countries in Table 3 in *Manuscript*. We present an overview of how many observations of which country we use in our analysis in Table S8.

2 Derivation of Short- and Long-Run Effects

The interpretation of the coefficients of equation (2) is percentage wise and we can separate the effect of lagged variables into a short- and a long-run effect. For example, a one-unit increase of UVI at time s affects the cumulative COVID-19 deaths $D_{t=s}$ via $\beta_{UVI,0}$ in the short-run. After one week, the increase of UVI affects $D_{t=s+1}$ firstly via $D_{t=s}$ and secondly via $\beta_{UV,1}$ because $D_{t=s+1} = D_{t=s}e^{\beta_{UV,1}}$ (partialling out the daily growth rate γ and keeping the control variables constant). Consequently, if the model consists of 5 lags, then the long-run effect will be reached after 5 weeks (see Table S1). Therefore, an increase of UVI by one-unit increases the cumulative COVID-19 deaths approximately by $(\beta_{UV,0} + \beta_{UV,1} + \beta_{UV,2} + \beta_{UV,3} + \beta_{UV,4} + \beta_{UV,5}) \times 100\%$ percent in the long-run (the exact value is $(e^{(\beta_{UV,0}+\beta_{UV,1}+\beta_{UV,2}+\beta_{UV,3}+\beta_{UV,4}+\beta_{UV,5})} - 1) \times 100\%$). In comparison, a permanent increase of UVI by one-unit increases the cumulative COVID-19 deaths approximately by $(\beta_{UV,0} + \beta_{UV,1} + \beta_{UV,2} + \beta_{UV,3} + \beta_{UV,4} + \beta_{UV,5}) \times 100\%$ each day.

3 Identification of Effect of Ultraviolet Index (UVI)

The key assumption that is required to identify a causal effect of UVI on the cumulative COVID-19 deaths is that $UVI_{i,t-s}$ is uncorrelated to $\epsilon_{i,t}$ at all points in time. This means that 1) past or future unexplained parts of $D_{i,t}$ must not affect $UVI_{i,t}$. These unexplained parts would appear in $\epsilon_{i,t}$ and be correlated with $UVI_{i,t-s}$ for some s. The key assumption requires further, that 2) there is no factor affecting $D_{i,t}$ which also influences $UVI_{i,t}$ in addition to country-specific time-constant factors or those variables which we include in the analysis. This additional factor would appear in $\epsilon_{i,t}$ and correlate with $UVI_{i,t-s}$ for some s. Both requirements are always satisfied, if UVI is randomly distributed after controlling for an appropriate set of control variables[1].

The first assumption is likely satisfied because the cumulative COVID-19 deaths cannot influence UVI. However, there have been structural changes with respect to the behaviour of individuals because the number of COVID-19 deaths likely influences them. Currently, individuals of regions where COVID-19 is present are less likely to go outside. However, going outside is a precondition for individuals to absorb UVB. Suppose the effect of UVI is β_{high} high for the first ten observations and β_{low} for the subsequent twenty observations, because individuals do not go outside and absorb UVB. Then, the estimate β_{est} recovers the weighted average of both effects, meaning that $\beta_{est} = \frac{1}{3}\beta_{high} + \frac{2}{3}\beta_{low}$, but would not be biased due to the behavioural change. We control for some changes in the behaviours, which may lead to more or less exposure to UVB radiation in two ways. Firstly, the set of weather variables partially controls for variations in individuals' exposure to UVB radiation because individuals are more likely to go outside on less cloudy or on days without rain. Secondly, we control for changes in individuals' exposure of UVB radiation induced by governmental measures (see our description in Table S9).

The second assumption could be violated by changes in the growth rates of the cumulative COVID-19 deaths with respect to countries over time. Such changes could be growth rates which are 1) country-specific and time-constant, 2) linearly time-varying but similar across countries, 3) non-linearly time-varying but similar across countries, and 4) linearly or non-linearly time-varying and country-specific. Such changes in the growth rates threaten a causal identification of the effect of UVI because UVI increases over our observation period as summer is coming closer (in the countries on the Northern hemisphere). Our main model specification isolates the effect of UVI from changes in the growth rates of type 1) and type 2) by partialling out any country-specific time-constant differences of growth rates and linear changes of growth rates with respect to time that are similar across countries. In our robustness checks we increase flexibility of the model to also capture country-specific linear

differences of growth rates with respect to time as well as some non-linear differences of growth rates with respect to time, which are either similar across countries or even country-specific.

The cloud and precipitation index could also violate condition 2). On the one hand, a high cloud coverage and precipitation today decrease the future number of infections because individuals are less likely to go outside and get infected today and die in future. On the other hand, both indices decrease UVI, because they absorb the radiation. Therefore, these two relationships could create an upward bias in the estimate of UVI. Consequently, controlling for the cloud as well as the precipitation index mitigates the bias and makes a causal identification more plausible.

The air quality could violate condition 2). The decrease in traffic because of COVID-19 over the time of our observation period leads to an improvement of the air quality and air quality is likely to reduce the cumulative COVID-19 deaths[2]. Because UVI increases over time, the air quality could be positively correlated with UVI. These relationships could cause an upwards bias in the estimate of UVI meaning that the true effect of UVI is lower that the estimate. Therefore, controlling for the visibility as a proxy variable for the air quality mitigates the bias and makes a causal identification more plausible.

A country-specific mutation of the virus over time could also violate assumption 2. If a mutation increased the cumulative COVID-19 deaths, then it could positively correlate with UVI because UVI also increased over the time of our observation period. This relationship would create an upward bias. Therefore, negative estimates of the effect of UVI can be considered conservative. Another threat to our identification is a potential systematic false reporting about the cumulative COVID-19 deaths. In the beginning of the crisis it seems likely that not all deaths were tested for COVID-19 (so that the reported number of COVID-19 deaths is smaller than the true value) while nowadays more deaths are tested and reported

as a COVID-19 death, even though not entirely caused by it (i.e., reported COVID-19 deaths is higher than true value). This positive correlation of measurement error with time would generate an upward bias of the estimate of the effect of UVI. Therefore, negative estimates can be considered conservative.

Country-specific governmental measures aimed at mitigating the spread of COVID-19 could also violate assumption 2. If such a measure decreases the number of COVID-19 deaths, then it will be negatively correlated with UVI because UVI increased over time. This relationship could create a downward bias of the estimate of the effect of UVI. Therefore, controlling for country-specific governmental measures mitigates this potential bias.

4 Model Selection to Identify the Effect of Ultraviolet Index (UVI) on the Cumulative COVID-19 Deaths

We estimate equation (2) up to a lag of 8 weeks and decided to choose models with 5 lags and all control variables as presented in Table S2. On the one hand, we did not find major changes with respect to the size and statistical significance of the estimate of UVI. On the other hand, including more lags increases the number of estimates but not the number of observations so that the accuracy of estimates decreases, which favours a more parsimonious model.

5 Robustness Checks

To examine the robustness of our results we change the dependent variable into the case-fatality-rate (CFR). CFR is defined as the cumulative COVID-19 deaths divided by the cumulative COVID-19 infections. Therefore, CFR of country i on day t is calculated as $\mathrm{CFR}_{i,t} = \frac{D_{i,t}}{I_{i,t}}$, where $I_{i,t}$ stands for the cumulative COVID-19 infections. It is a common measure to assess the severity of diseases because a high CFR leads to a high number of cumulative COVID-19 deaths. Its advantage to the cumulative COVID-19 deaths is that it relates the cumulative number of deaths to the cumulative number of infections for a disease. Therefore, it helps to isolate the effect of UVI on cumulative COVID-19 deaths from its effect

on cumulative COVID-19 infections. Provided that the cumulative COVID-19 infections follow the same model structure as outlined in equation (2), we can express $I_{i,t}$ via an exponential growth model in equation (3):

$$I_{i,t} = I_{i,t-1} \times e^{\gamma_I + \sum_{j=0}^{5}[UVI_{i,t-j}\beta_{I,UVI,j} + C_{i,t-j}\beta_{I,C,j}] + FI_{i,t}\beta_{I,FI} + u_{I,i} + \epsilon_{I,i,t}} \quad (3)$$

The interpretation of the coefficients and variables is essentially the same as in equation (2) but relates to the cumulative COVID-19 infections rather than deaths. Dividing equation (1) by equation (3) leads to equation (4).

$$CFR_{i,t} = CFR_{i,t-1} \times e^{\gamma_{CFR} + \sum_{j=0}^{5}[UVI_{i,t-j}\beta_{CFR,UVI,j} + C_{i,t-j}\beta_{CFR,C,j}] + FI_{i,t}\beta_{CFR,FI} + u_{CFR,i} + \epsilon_{CFR,i,t}} \quad (4)$$

After applying the same transformation on equation (4) to derive equation (2) we get the estimable equation (5).

$$\Delta \ln(\ddot{CFR}_{i,t}) = \sum_{j=0}^{5} [\ddot{UVI}_{i,t-j}\beta_{CFR,UVI,j} + \ddot{C}_{i,t-j}\beta_{CFR,C,j}] + \ddot{FI}_{i,t}\beta_{CFR,FI} + \ddot{\epsilon}_{CFR,i,t} \quad (5)$$

Every coefficient of equation (5) shows the effect of UVI or the effect of a control variable on CFR. For example, $\beta_{CFR,UVI,j}$ stands for the effect of UVI lagged by j weeks on CFR. Moreover, $\beta_{CFR,UVI,j}$ reflects the difference of the effect of UVI on the cumulative COVID-19 deaths and infections, because $\beta_{CFR,UVI,j} = \beta_{UVI,j} - \beta_{I,UVI,j}$. This relationship also holds for $\beta_{CFR,C,j}$ and $\beta_{CFR,FI}$.We expect to find smaller effect sizes for UVI on CFR. On the one hand, we expect UVI to decrease the cumulative COVID-19 deaths. On the other hand, we expect UVI to decrease the cumulative COVID-19 infections. The reason is not that fewer people get infected but rather that the infections are less severe which makes it less likely that people get themselves tested. One concern when using the observed case fatality rate CFR^{obs} that is obtained during an epidemic, is that it likely understates the true case fatality rate CFR^{true}. Note, however, that the model is robust to a miss-reported value of CFR^{true} as long as the error is multiplicative and time-constant. Suppose the observed case fatality rate is

$$CFR_{i,t}^{obs} = CFR_{i,t}^{true} \times \vartheta_{CFR} \quad (6)$$

where ϑ_{CFR} represents the multiplicative and time-constant error. This relationship could be the result of miss-reported cumulative COVID-19 deaths and infections, if there are time-constant errors for the cumulative COVID-19 deaths and infections, because

$$CFR_{i,t}^{obs} = \frac{D_{i,t}^{true} \times \vartheta_D}{I_{i,t}^{true} \times \vartheta_I} = \frac{D_{i,t}^{true}}{I_{i,t}^{true}} \times \vartheta_{CFR} \quad (7)$$

where ϑ_D and ϑ_I represent the multiplicative and time-constant error for the cumulative COVID-19 deaths and infections, respectively. If, for example, the true cumulative COVID-19 deaths at day t are always 10% higher than the observed one, then this 10% difference represents a multiplicative and time-constant error.

If the multiplicative errors of cumulative COVID-19 deaths and infections are not time-constant but grow linearly over time, then the multiplicative error of CFR will still be time constant:

$$CFR_{i,t}^{obs} = \frac{D_{i,t}^{true} \times \vartheta_{D,t}}{I_{i,t}^{true} \times \vartheta_{I,t}} = \frac{D_{i,t}^{true} \times \vartheta_{D,0} \times \rho_D t}{I_{i,t}^{true} \times \vartheta_{I,0} \times \rho_I t} = \frac{D_{i,t}^{true} \times \vartheta_{D,0} \times \rho_D}{I_{i,t}^{true} \times \vartheta_{I,0} \times \rho_I} = \frac{D_{i,t}^{true} \times \vartheta_{D,*}}{I_{i,t}^{true} \times \vartheta_{I,*}} = \frac{D_{i,t}^{true}}{I_{i,t}^{true}} \times \vartheta_{CFR,*} \quad (8)$$

where $\vartheta_{D,t}$ and $\vartheta_{I,t}$ represent the multiplicative error for the cumulative COVID-19 deaths and infections, respectively, which could grow linearly over time. For example, if the true cumulative Covid-19 deaths or infections are first underreported and later over-reported, then ρ_D or ρ_I will be positive and $\vartheta_{D,t}$ as well as $\vartheta_{I,t}$ will first be negative and later (i.e., with larger values for t) become positive.

Even in the presence of those two forms of measurement-error outlined in equations (7) and (8), our statistical analysis is able to identify the effect of UVI on CFR.

The four aforementioned changes in growth rates of cumulative COVID-19 deaths with respect to time threaten a causal identification of the effect of UVI. Therefore, in addition to controlling for time-constant country-specific changes of growth rates as well as linear changes of growth rates that are similar across countries, we increase the flexibility of our main model specification. The more flexible model isolates the effect of UVI from time trends by allowing time to affect the growth rates of all countries linearly or non-linearly in the same or in a country-specific way. Essentially, in addition to $FI_{i,t}$ we also include $(FI_{i,t})^2$ and $e^{FI_{i,t}}$ into the model, and we interact each of the variables $FI_{i,t}$, $(FI_{i,t})^2$ and $e^{FI_{i,t}}$ with 152 dummy variables, one for each country, to allow for country-specific linear and non-linear time effects.

In addition, we address in our robustness checks that governments implemented different measures at different points in time to reduce the spread of COVID-19. Hale et al. (2020) collected data on such governmental measures over time[3]. We use 8 of their variables on the different indicators of governmental measures such as school closing, workplace closing, cancellation of public events, restrictions on gathering size, closure of public transport, stay at home requirements, restrictions on internal movement, and restrictions on international travel. Similarly to the control variables of equation (1), we add those 8 variables and their corresponding weekly lags to our model. Table S9 and Table S10 outline the results of the robustness checks. Because Hale et al. (2020) do not cover all countries of our original data set, the number of countries shrink from 152 to 136[3].

6 Derivation of Equation (2)

Start from equation (1).

$$D_{i,t} = D_{i,t-1} \times e^{\gamma + \sum_{j=0}^{5}[UVI_{i,t-j}\beta_{UVI,j} + C_{i,t-j}\beta_{C,j}] + FI_{i,t}\beta_{FI} + u_i + \epsilon_{i,t}} \quad (1)$$

Taking the natural logarithm leads to equation (1.1).

$$\ln(D_{i,t}) = \ln(D_{i,t-1}) + \gamma + \sum_{j=0}^{5}\left[UVI_{i,t-j}\beta_{UVI,j} + C_{i,t-j}\beta_{C,j}\right] + FI_{i,t}\beta_{FI} + u_i + \epsilon_{i,t} \qquad (1.1)$$

Deducting $\ln(D_{i,t-1})$ leads to equation (1.2)

$$\Delta\ln(D_{i,t}) = \ln(D_{i,t}) - \ln(D_{i,t-1}) = \gamma + \sum_{j=0}^{5}\left[UVI_{i,t-j}\beta_{UVI,j} + C_{i,t-j}\beta_{C,j}\right] + FI_{i,t}\beta_{FI} + u_i + \epsilon_{i,t} \qquad (1.2)$$

The average version of the left-hand side and right-hand side of equation (1.2) across time is given by equation (1.3) and (1.4), respectively.

$$\overline{\Delta\ln(D_{i,t})} = \frac{1}{T}\sum_{s=1}^{T}\Delta\ln(D_{i,s}) \qquad (1.3)$$

$$\gamma + \sum_{j=0}^{5}\left[\overline{UVI}_{i,t-j}\beta_{UVI,j} + \bar{C}_{i,t-j}\beta_{C,j}\right] + \overline{FI}_{i,t}\beta_{FI} + \bar{u}_i + \bar{\epsilon}_{i,t} = \frac{1}{T}\sum_{s=1}^{T}\left[\gamma + \sum_{j=0}^{5}\left[UVI_{i,t-j}\beta_{UVI,j} + C_{i,t-j}\beta_{C,j}\right] + FI_{i,t}\beta_{FI} + u_i + \epsilon_{i,t}\right] \qquad (1.4)$$

Deducting equation (1.3) and (1.4) from equation (1.2) leads to equation (2).

$$\Delta\ln(\ddot{D}_{i,t}) = \sum_{j=0}^{5}\left[U\ddot{V}I_{i,t-j}\beta_{UVI,j} + \ddot{C}_{i,t-j}\beta_{C,j}\right] + \ddot{F}I_{i,t}\beta_{FI} + \ddot{\epsilon}_{i,t} \qquad (2)$$

7 Identification of Daily Growth Rate γ

Instead of demeaning equation (1) we can assume that u_i is uncorrelated to all explanatory variables such that we can estimate a random effects model. Under these more restrictive assumptions, γ is identified. The results are provided in Table S4. The estimated daily growth rate increases COVID-19 deaths by 62% [$e^{0.4823}$-1]. A robust Hausman test to assess the plausibility of the additional assumptions required to identify γ is not rejected. Therefore, we

can use a random effects model . Nevertheless, the estimate needs to be interpreted with caution because the magnitude lacks theoretical plausibility.

8 Extended Regression Results (Fixed Effects Analysis)

Table S5, S6, and S7 outline the estimation results of our main model up to 8 weeks lagged for different sets of control variables. The estimates do not change substantially after we include five or more lags of UVI or the control variables. We find that the model with five lags is favourable and we use this model in our main analysis.

9 Supplementary Tables

Table S1: Short- and long-run effects of a unit increase in Ultraviolet Index (UVI)

Weeks passed by	Change of D_{t+s}
0	$\beta_{UV,0} \times 100\%$
1	$(\beta_{UV,0} + \beta_{UV,1}) \times 100\%$
2	$(\beta_{UV,0} + \beta_{UV,1} + \beta_{UV,2}) \times 100\%$
3	$(\beta_{UV,0} + \beta_{UV,1} + \beta_{UV,2} + \beta_{UV,3}) \times 100\%$
4	$(\beta_{UV,0} + \beta_{UV,1} + \beta_{UV,2} + \beta_{UV,3} + \beta_{UV,4}) \times 100\%$
5 or more	$(\beta_{UV,0} + \beta_{UV,1} + \beta_{UV,2} + \beta_{UV,3} + \beta_{UV,4} + \beta_{UV,5}) \times 100\%$

Table S2: Robustness check for linear and quadratic time trends

	Model 3	Model 4	Model 5
Dependent Variables	COVID-19 Deaths	COVID-19 Deaths	COVID-19 Deaths
L0.UVI	-0.002 (-1.53)	-0.000 (-0.24)	0.000 (0.05)
L1.UVI	0.000 (0.08)	0.002 (0.92)	0.001 (0.43)
L2.UVI	-0.001 (-0.97)	-0.000 (-0.28)	-0.001 (-0.45)
L3.UVI	-0.002 (-1.36)	-0.002 (-1.10)	-0.002 (-1.33)
L4.UVI	-0.004* (-2.08)	-0.003+ (-1.94)	-0.004* (-2.02)
L5.UVI	-0.002 (-1.29)	-0.002 (-0.99)	-0.002 (-1.13)
Long-Run Coefficient	-0.012** (F: 8.76)	-0.006 (F: 2.45)	-0.007* (F: 4.04)
**Control variables**			
Time Trend	Linear and square	Linear (country-specific)	Linear and square (country-specific)
Country Fixed Effects	Yes	Yes	Yes
Precipitation index	Yes	Yes	Yes
Cloud index	Yes	Yes	Yes
Ozone level	Yes	Yes	Yes
Visibility Level	Yes	Yes	Yes
Humidity level	Yes	Yes	Yes
Temperature (min and max)	Yes	Yes	Yes
Number of Estimates	50 (+152 FE)	48 (+152 FE + 152 TSCE)	48 (+152 FE + 304 TSCE)
Number of Observations	6,524	6,524	6,524
Number of Countries	152	152	152
R-squared Within	14.00%	19.01%	24.04%

Note: +: p < 0.10, *: p < 0.05, **: p < 0.01. t-statistics based on robust standard errors in parentheses. F-statistic for long-run coefficient in parentheses. L0.UVI stands for the effect of UVI at time t on the cumulative number of COVID-19 deaths at the same time, whereas L1.UVI, L2.UVI, L3.UVI, L4.UVI and L5.UVI stand for the effect of UVI lagged by one, two, three, four or five weeks respectively. FE stands for country fixed-effects, TCSE stands for time country-specific effects.

Table S3: Robustness check for exponential, linear, quadratic time trends

	Model 6	Model 7	Model 8
Dependent Variables	COVID-19 Deaths	COVID-19 Deaths	COVID-19 Deaths
L0.UVI	-0.003 (-1.60)	-0.000 (-0.12)	-0.000 (-0.25)
L1.UVI	0.000 (0.01)	0.002 (0.91)	0.001 (0.44)
L2.UVI	-0.001 (-0.95)	-0.001 (-0.42)	-0.001 (-0.53)
L3.UVI	-0.002 (-1.28)	-0.002 (-1.06)	-0.002 (-1.39)
L4.UVI	-0.004* (-2.06)	-0.003+ (-1.82)	-0.004+ (-1.91)
L5.UVI	-0.002 (-1.25)	-0.002 (-1.00)	-0.002 (-1.15)
Long-Run Coefficient	-0.012** (F: 8.53)	-0.006 (F2.27)	-0.008* (F: 4.69)
**Control variables**			
Time Trend	Linear and exponential	Linear and exponential (country-specific)	Linear, square and exponential (country-specific)
Country Fixed-Effects	Yes	Yes	Yes
Precipitation index	Yes	Yes	Yes
Cloud index	Yes	Yes	Yes
Ozone level	Yes	Yes	Yes
Visibility Level	Yes	Yes	Yes
Humidity level	Yes	Yes	Yes
Temperature (min and max)	Yes	Yes	Yes
Number of Estimates	50 (+152 FE)	48 (+152 FE + 304 TSCE)	48 (+152 FE + 456 TSCE)
Number of Observations	6,524	6,524	6,524
Number of Countries	152	152	152
R-squared Within	13.78%	19.64%	25.00%

Note: +: p < 0.10, *: p < 0.05, **: p < 0.01. t-statistics based on robust standard errors in parentheses. F-statistic for long-run coefficient in parentheses. L0.UVI stands for the effect of UVI at time t on the cumulative number of COVID-19 deaths at the same time, whereas L1.UVI, L2.UVI, L3.UVI, L4.UVI and L5.UVI stand for the effect of UVI lagged by one, two, three, four or five weeks respectively. FE stands for country fixed-effects, TCSE stands for time country-specific effects.

Table S4: Identification of daily growth rate γ

	Model 9
γ	0.4823
L0.UVI	-0.002 (-0.93)
L1.UVI	0.000 (0.17)
L2.UVI	-0.001 (-0.99)
L3.UVI	-0.002 (-1.57)
L4.UVI	-0.004* (-2.45)
L5.UVI	-0.004* (-2.14)
Long-Run Coefficient	-0.013*** (F: 13.63)
**Control variables**	
Time Trend	Linear
Precipitation index	Yes
Cloud index	Yes
Ozone level	Yes
Visibility Level	Yes
Humidity level	Yes
Temperature (min and max)	Yes
Number of Estimates	50
Number of Observations	6,524
Number of Countries	152
R-squared within	13.03%
p-value of Robust Hausman-test	0.6661

Note: +: $p < 0.10$, *: $p < 0.05$, **: $p < 0.01$. t-statistics based on robust standard errors in parentheses. F-statistic for long-run coefficient in parentheses. Robust Hausman-test based on 2,000 clustered bootstrap replications. L0.UVI stands for the effect of UVI at time t on the cumulative number of COVID-19 deaths at the same time, whereas L1.UVI, L2.UVI, L3.UVI, L4.UVI and L5.UVI stand for the effect of UVI lagged by one, two, three, four or five weeks respectively. FE stands for country fixed-effects.

Table S5: Estimation of models with different lags without control variables

	Model A1.1	Model A1.2	Model A1.3	Model A1.4	Model A1.5	Model A1.6	Model A1.7	Model A1.8	Model A1.9
Time Since First Infection	-0.003*** (-11.53)	-0.003*** (-11.48)	-0.003*** (-11.33)	-0.003*** (-11.05)	-0.003*** (-11.95)	-0.004*** (-13.76)	-0.004*** (-14.80)	-0.004*** (-16.61)	-0.004*** (-17.04)
L0.UVI	-0.001 (-1.21)	-0.001 (-1.22)	-0.001 (-1.19)	-0.001 (-1.10)	-0.001 (-1.15)	-0.001 (-1.04)	-0.001 (-0.88)	-0.001 (-0.88)	-0.001 (-0.97)
L1.UVI		0.000 (0.04)	0.000 (0.09)	0.000 (0.14)	0.000 (0.11)	0.000 (0.34)	0.001 (0.50)	0.001 (0.47)	0.001 (0.95)
L2.UVI			-0.002 (-1.51)	-0.001 (-1.40)	-0.001 (-1.31)	-0.001 (-1.18)	-0.001 (-0.97)	-0.001 (-0.74)	-0.001 (-0.54)
L3.UVI				-0.003** (-2.98)	-0.003** (-3.01)	-0.003** (-2.91)	-0.003** (-2.82)	-0.003** (-2.83)	-0.003** (-2.62)
L4.UVI					-0.001 (-0.84)	-0.001 (-0.98)	-0.001 (-1.00)	-0.001 (-1.00)	-0.001 (-1.13)
L5.UVI						-0.000 (-0.28)	-0.000 (-0.43)	-0.001 (-0.51)	-0.001 (-0.57)
L6.UVI							-0.000 (-0.17)	-0.000 (-0.32)	-0.000 (-0.09)
L7.UVI								-0.002* (-2.16)	-0.002* (-2.11)
L8.UVI									-0.001 (-0.93)
Constant	0.261*** (15.24)	0.261*** (14.02)	0.268*** (13.49)	0.282*** (13.93)	0.291*** (14.13)	0.297*** (14.94)	0.299*** (15.10)	0.313*** (15.65)	0.312*** (15.39)
Number of Observations	6,588	6,588	6,588	6,586	6,568	6,524	6,468	6,381	6,223
Number of States	152	152	152	152	152	152	152	152	152
Adjusted R-squared	0.116	0.116	0.116	0.117	0.121	0.125	0.128	0.130	0.130

Note: +: p < 0.10, *: p < 0.05, **: p < 0.01. t-statistics based on robust standard errors in parentheses. L0.UVI stands for the effect of UVI at time t on the cumulative number of COVID-19 deaths at the same time, whereas L1.UVI, L2.UVI and L3.UVI (etc.) stand for the effect of UVI lagged by one, two, or three (etc.) weeks respectively. The same is true for the other variables.

Table S6: Estimation of models with different lags with control variables affecting ultraviolet index

	Model A2.1	Model A2.2	Model A2.3	Model A2.4	Model A2.5	Model A2.6	Model A2.7	Model A2.8	Model A2.9
Time Since First Infection	-0.003*** (-11.31)	-0.003*** (-11.20)	-0.003*** (-11.13)	-0.003*** (-11.16)	-0.003*** (-12.12)	-0.003*** (-13.38)	-0.004*** (-14.34)	-0.004*** (-15.10)	-0.004*** (-14.57)
L0.UVI	-0.002 (-1.42)	-0.002 (-1.34)	-0.002 (-1.25)	-0.002 (-1.24)	-0.002 (-1.34)	-0.002 (-1.24)	-0.002 (-1.18)	-0.002 (-1.06)	-0.002 (-0.96)
L1.UVI		0.000 (0.27)	0.001 (0.32)	0.001 (0.34)	0.001 (0.32)	0.001 (0.43)	0.001 (0.39)	0.001 (0.51)	0.001 (0.72)
L2.UVI			-0.000 (-0.29)	-0.000 (-0.22)	-0.000 (-0.21)	-0.000 (-0.07)	-0.000 (-0.18)	0.000 (0.28)	0.000 (0.23)
L3.UVI				-0.001 (-0.39)	-0.000 (-0.18)	-0.001 (-0.44)	-0.001 (-0.39)	-0.001 (-0.44)	-0.000 (-0.17)
L4.UVI					-0.002 (-1.19)	-0.002 (-1.34)	-0.002 (-1.44)	-0.002 (-1.48)	-0.003+ (-1.81)
L5.UVI						-0.001 (-0.68)	-0.001 (-0.70)	-0.002 (-1.08)	-0.002 (-1.02)
L6.UVI							0.001 (0.72)	0.001 (0.64)	0.001 (0.57)
L7.UVI								-0.002 (-1.46)	-0.002 (-1.38)
L8.UVI									-0.000 (-0.12)
L0.CLOUD	-0.014 (-1.16)	-0.014 (-1.12)	-0.013 (-1.01)	-0.014 (-1.12)	-0.016 (-1.25)	-0.015 (-1.17)	-0.014 (-1.08)	-0.012 (-0.96)	-0.012 (-0.92)
L1.CLOUD		-0.009 (-0.75)	-0.007 (-0.62)	-0.007 (-0.59)	-0.006 (-0.56)	-0.008 (-0.72)	-0.009 (-0.83)	-0.006 (-0.51)	-0.007 (-0.60)
L2.CLOUD			0.002 (0.22)	0.003 (0.31)	0.004 (0.34)	0.004 (0.32)	0.001 (0.08)	0.006 (0.52)	0.005 (0.45)
L3.CLOUD				0.014 (1.35)	0.017 (1.63)	0.012 (1.18)	0.012 (1.24)	0.011 (1.13)	0.016+ (1.75)
L4.CLOUD					-0.004 (-0.33)	-0.005 (-0.44)	-0.004 (-0.30)	-0.004 (-0.36)	-0.007 (-0.65)
L5.CLOUD						-0.006 (-0.55)	-0.003 (-0.27)	-0.010 (-0.89)	-0.007 (-0.65)
L6.CLOUD							0.008 (0.76)	0.009 (0.93)	0.008 (0.86)
L7.CLOUD								-0.002 (-0.17)	0.002 (0.16)
L8.CLOUD									0.013 (1.46)
L0.PRECIP	-0.016+ (-1.73)	-0.015 (-1.63)	-0.015 (-1.65)	-0.013 (-1.46)	-0.011 (-1.23)	-0.011 (-1.16)	-0.010 (-1.12)	-0.008 (-0.87)	-0.007 (-0.73)
L1.PRECIP		0.019+ (1.83)	0.019+ (1.79)	0.019+ (1.80)	0.018+ (1.77)	0.023* (2.20)	0.020* (2.10)	0.017+ (1.78)	0.016+ (1.80)
L2.PRECIP			0.005 (0.45)	0.003 (0.28)	0.003 (0.24)	0.004 (0.40)	0.006 (0.51)	0.006 (0.54)	0.006 (0.53)
L3.PRECIP				0.018 (1.54)	0.018 (1.54)	0.019+ (1.67)	0.018 (1.56)	0.018 (1.64)	0.016 (1.35)
L4.PRECIP					-0.009 (-1.02)	-0.008 (-1.01)	-0.010 (-1.24)	-0.009 (-1.08)	-0.009 (-1.05)
L5.PRECIP						-0.005 (-0.57)	-0.006 (-0.72)	-0.005 (-0.57)	-0.013 (-1.54)
L6.PRECIP							0.001 (0.16)	-0.002 (-0.26)	-0.004 (-0.45)
L7.PRECIP								0.008 (0.87)	0.004 (0.46)

L8.PRECIP									0.007 (0.69)
L0.OZONE	0.000+ (1.91)	0.000+ (1.77)	0.000+ (1.77)	0.000 (1.46)	0.000 (1.17)	0.000 (1.13)	0.000 (1.30)	0.000 (1.24)	0.000 (1.48)
L1.OZONE		0.000 (0.76)	0.000 (0.56)	0.000 (0.41)	0.000 (0.58)	0.000 (0.69)	0.000 (1.36)	0.000 (1.38)	0.000 (1.62)
L2.OZONE			0.000 (0.49)	0.000 (0.33)	-0.000 (-0.01)	-0.000 (-0.10)	-0.000 (-0.36)	-0.000 (-0.57)	-0.000 (-1.17)
L3.OZONE				-0.000 (-0.13)	0.000 (0.08)	0.000 (0.15)	0.000 (0.56)	0.000 (0.58)	0.000 (0.18)
L4.OZONE					-0.000 (-0.67)	-0.000 (-0.73)	-0.000 (-0.84)	-0.000 (-1.07)	-0.000 (-1.23)
L5.OZONE						-0.000 (-0.34)	-0.000 (-0.62)	-0.000 (-0.27)	0.000 (0.61)
L6.OZONE							0.000* (2.18)	0.000+ (1.97)	0.000 (1.36)
L7.OZONE								-0.000 (-0.84)	-0.000 (-0.81)
L8.OZONE									-0.000* (-2.10)
L0.VISIBILITY	-0.003* (-2.29)	-0.003* (-2.26)	-0.003* (-2.30)	-0.003* (-2.26)	-0.003* (-2.24)	-0.003* (-2.03)	-0.002 (-1.61)	-0.002 (-1.57)	-0.003+ (-1.84)
L1.VISIBILITY		0.001 (0.54)	0.001 (0.57)	0.000 (0.44)	0.001 (0.58)	0.001 (1.28)	0.001 (1.11)	0.001 (1.11)	0.001 (1.22)
L2.VISIBILITY			-0.001 (-0.77)	-0.001 (-0.97)	-0.001 (-0.85)	-0.001 (-0.70)	-0.001 (-0.54)	-0.000 (-0.39)	-0.000 (-0.20)
L3.VISIBILITY				0.000 (0.18)	0.000 (0.28)	0.001 (0.46)	0.001 (0.54)	0.000 (0.42)	0.001 (0.92)
L4.VISIBILITY					-0.000 (-0.01)	-0.000 (-0.09)	-0.000 (-0.02)	0.000 (0.11)	0.000 (0.23)
L5.VISIBILITY						-0.001 (-0.62)	-0.000 (-0.47)	-0.001 (-0.59)	-0.001 (-1.08)
L6.VISIBILITY							0.000 (0.09)	-0.000 (-0.23)	0.000 (0.02)
L7.VISIBILITY								-0.001 (-0.55)	-0.001 (-0.54)
L8.VISIBILITY									0.001 (1.06)
Constant	0.242*** (4.22)	0.208* (2.38)	0.209+ (1.81)	0.227 (1.58)	0.268 (1.65)	0.290+ (1.87)	0.193 (1.22)	0.255+ (1.67)	0.293+ (1.71)
Number of Observations	6,588	6,588	6,588	6,586	6,568	6,524	6,468	6,381	6,223
Number of States	152	152	152	152	152	152	152	152	152
Adjusted R-squared	0.118	0.119	0.120	0.122	0.126	0.131	0.134	0.136	0.137

Note: +: p < 0.10, *: p < 0.05, **: p < 0.01. t-statistics based on robust standard errors in parentheses. L0.UVI stands for the effect of UVI at time t on the cumulative number of COVID-19 deaths at the same time, whereas L1.UVI, L2.UVI and L3.UVI (etc.) stand for the effect of UVI lagged by one, two, or three (etc.) weeks respectively. The same is true for the other variables.

Table S7: Estimation of models with different lags with all control variables

	Model A3.1	Model A3.2	Model A3.3	Model A3.4	Model A3.5	Model A3.6	Model A3.7	Model A3.8	Model A3.9
Time Since First Infection	-0.003*** (-11.03)	-0.003*** (-10.67)	-0.003*** (-10.83)	-0.004*** (-10.91)	-0.004*** (-11.73)	-0.004*** (-13.31)	-0.004*** (-14.27)	-0.004*** (-14.73)	-0.004*** (-14.24)
L0.UVI	-0.003 (-1.47)	-0.002 (-1.38)	-0.003 (-1.44)	-0.003 (-1.50)	-0.003 (-1.56)	-0.002 (-1.53)	-0.003 (-1.56)	-0.002 (-1.49)	-0.002 (-1.34)
L1.UVI		0.000 (0.18)	0.000 (0.10)	0.000 (0.00)	-0.000 (-0.01)	0.000 (0.02)	-0.000 (-0.03)	0.000 (0.09)	0.001 (0.38)
L2.UVI			-0.002 (-0.98)	-0.002 (-1.04)	-0.002 (-1.02)	-0.002 (-1.03)	-0.002 (-1.14)	-0.001 (-0.72)	-0.001 (-0.75)
L3.UVI				-0.002 (-1.25)	-0.001 (-1.02)	-0.002 (-1.29)	-0.002 (-1.31)	-0.002 (-1.34)	-0.001 (-1.01)
L4.UVI					-0.003+ (-1.80)	-0.003* (-2.03)	-0.003* (-2.05)	-0.003* (-2.10)	-0.004* (-2.30)
L5.UVI						-0.002 (-1.23)	-0.002 (-1.15)	-0.002 (-1.49)	-0.002 (-1.24)
L6.UVI							0.000 (0.25)	0.000 (0.26)	0.001 (0.47)
L7.UVI								-0.003* (-2.01)	-0.002 (-1.50)
L8.UVI									-0.001 (-0.96)
L0.CLOUD	-0.015 (-1.23)	-0.015 (-1.14)	-0.013 (-1.01)	-0.015 (-1.10)	-0.015 (-1.08)	-0.012 (-0.91)	-0.014 (-1.03)	-0.014 (-1.04)	-0.013 (-0.96)
L1.CLOUD		-0.011 (-1.00)	-0.011 (-0.97)	-0.012 (-1.03)	-0.011 (-1.00)	-0.013 (-1.15)	-0.013 (-1.14)	-0.009 (-0.77)	-0.011 (-0.94)
L2.CLOUD			-0.012 (-1.07)	-0.012 (-1.02)	-0.011 (-0.95)	-0.011 (-0.94)	-0.011 (-0.97)	-0.007 (-0.66)	-0.009 (-0.78)
L3.CLOUD				0.009 (0.82)	0.012 (1.10)	0.008 (0.73)	0.006 (0.62)	0.007 (0.65)	0.012 (1.21)
L4.CLOUD					-0.017 (-1.29)	-0.017 (-1.35)	-0.012 (-1.04)	-0.012 (-1.06)	-0.013 (-1.21)
L5.CLOUD						-0.017 (-1.54)	-0.011 (-1.01)	-0.016 (-1.50)	-0.012 (-1.11)
L6.CLOUD							0.003 (0.28)	0.007 (0.62)	0.008 (0.72)
L7.CLOUD								-0.009 (-1.00)	-0.003 (-0.30)
L8.CLOUD									0.002 (0.21)
L0.PRECIP	-0.016 (-1.53)	-0.015 (-1.40)	-0.017 (-1.61)	-0.016 (-1.54)	-0.014 (-1.34)	-0.013 (-1.20)	-0.014 (-1.27)	-0.011 (-1.00)	-0.008 (-0.76)
L1.PRECIP		0.014 (1.17)	0.014 (1.14)	0.013 (1.09)	0.014 (1.11)	0.017 (1.43)	0.015 (1.30)	0.011 (0.95)	0.007 (0.66)
L2.PRECIP			0.007 (0.51)	0.007 (0.52)	0.007 (0.53)	0.009 (0.76)	0.012 (0.98)	0.010 (0.79)	0.008 (0.65)
L3.PRECIP				0.020 (1.59)	0.021 (1.63)	0.024+ (1.91)	0.024+ (1.94)	0.025* (2.03)	0.020 (1.58)
L4.PRECIP					-0.015 (-1.51)	-0.012 (-1.28)	-0.014 (-1.43)	-0.010 (-1.10)	-0.010 (-1.06)
L5.PRECIP						-0.013 (-1.30)	-0.012 (-1.15)	-0.008 (-0.80)	-0.014 (-1.58)
L6.PRECIP							-0.008 (-0.85)	-0.009 (-1.01)	-0.009 (-1.03)

L7.PRECIP								-0.001 (-0.14)	-0.007 (-0.74)
L8.PRECIP									-0.000 (-0.04)
L0.OZONE	0.000+ (1.87)	0.000 (1.64)	0.000 (1.64)	0.000 (1.40)	0.000 (1.27)	0.000 (1.23)	0.000 (1.41)	0.000 (1.50)	0.000 (1.51)
L1.OZONE		0.000 (0.85)	0.000 (0.43)	0.000 (0.03)	0.000 (0.10)	0.000 (0.52)	0.000 (1.34)	0.000 (1.47)	0.000* (1.99)
L2.OZONE			0.000 (1.07)	0.000 (0.83)	0.000 (0.46)	0.000 (0.21)	0.000 (0.18)	0.000 (0.03)	-0.000 (-0.37)
L3.OZONE				0.000 (0.32)	0.000 (0.57)	0.000 (0.58)	0.000 (1.14)	0.000 (1.33)	0.000 (0.64)
L4.OZONE					-0.000 (-0.36)	-0.000 (-0.32)	-0.000 (-0.88)	-0.000 (-1.08)	-0.000 (-0.96)
L5.OZONE						-0.000 (-0.20)	-0.000 (-0.45)	-0.000 (-0.29)	0.000 (0.38)
L6.OZONE							0.000* (2.51)	0.000* (2.44)	0.000 (1.37)
L7.OZONE								-0.000 (-0.28)	-0.000 (-0.04)
L8.OZONE									-0.000 (-1.43)
L0.VISIBILITY	-0.003* (-2.25)	-0.003* (-2.22)	-0.003* (-2.31)	-0.003* (-2.32)	-0.003* (-2.34)	-0.003* (-2.16)	-0.002+ (-1.69)	-0.002+ (-1.72)	-0.003* (-2.04)
L1.VISIBILITY		0.001 (0.49)	0.000 (0.38)	0.000 (0.25)	0.000 (0.41)	0.001 (0.89)	0.001 (0.64)	0.001 (0.71)	0.001 (0.71)
L2.VISIBILITY			-0.002 (-1.28)	-0.002 (-1.59)	-0.002 (-1.51)	-0.002 (-1.45)	-0.002 (-1.27)	-0.001 (-1.11)	-0.001 (-0.77)
L3.VISIBILITY				-0.001 (-0.57)	-0.001 (-0.50)	-0.000 (-0.39)	-0.000 (-0.31)	-0.001 (-0.41)	0.000 (0.10)
L4.VISIBILITY					-0.000 (-0.20)	-0.000 (-0.35)	-0.000 (-0.14)	0.000 (0.03)	0.000 (0.25)
L5.VISIBILITY						-0.001 (-1.03)	-0.001 (-0.92)	-0.001 (-1.05)	-0.001 (-1.43)
L6.VISIBILITY							0.000 (0.06)	-0.000 (-0.32)	0.000 (0.05)
L7.VISIBILITY								-0.001 (-0.66)	-0.001 (-0.60)
L8.VISIBILITY									0.001 (0.81)
L0.HUMIDITY	0.000 (0.02)	-0.001 (-0.02)	0.003 (0.10)	0.005 (0.18)	0.003 (0.12)	0.000 (0.01)	0.006 (0.25)	0.007 (0.26)	0.003 (0.14)
L1.HUMIDITY		0.024 (0.89)	0.020 (0.75)	0.021 (0.79)	0.020 (0.74)	0.018 (0.66)	0.021 (0.76)	0.028 (1.03)	0.043+ (1.66)
L2.HUMIDITY			-0.036 (-1.24)	-0.045 (-1.55)	-0.047 (-1.63)	-0.049+ (-1.74)	-0.054+ (-1.93)	-0.054+ (-1.96)	-0.050+ (-1.85)
L3.HUMIDITY				0.007 (0.29)	0.005 (0.21)	-0.005 (-0.20)	-0.010 (-0.42)	-0.016 (-0.66)	-0.012 (-0.51)
L4.HUMIDITY					0.025 (1.03)	0.018 (0.76)	0.008 (0.36)	0.005 (0.23)	0.001 (0.04)
L5.HUMIDITY						0.004 (0.19)	-0.005 (-0.22)	-0.016 (-0.76)	-0.021 (-1.00)
L6.HUMIDITY							0.039+ (1.68)	0.038 (1.56)	0.032 (1.39)
L7.HUMIDITY								0.024 (1.04)	0.033 (1.37)
L8.HUMIDITY									0.032 (1.46)

L0.TEMP_MAX	-0.000 (-0.10)	-0.000 (-0.03)	-0.000 (-0.08)	0.000 (0.01)	0.000 (0.07)	0.000 (0.19)	0.000 (0.09)	0.000 (0.10)	0.000 (0.34)
L1.TEMP_MAX		0.000 (0.09)	0.000 (0.18)	0.000 (0.09)	0.000 (0.01)	0.000 (0.11)	0.001 (0.70)	0.001 (0.94)	0.001 (1.14)
L2.TEMP_MAX			-0.002+ (-1.96)	-0.002+ (-1.84)	-0.002+ (-1.83)	-0.002 (-1.63)	-0.002 (-1.24)	-0.002 (-1.65)	-0.002+ (-1.76)
L3.TEMP_MAX				0.001 (1.25)	0.001 (1.30)	0.001 (1.26)	0.001 (1.03)	0.001 (0.95)	0.001 (0.56)
L4.TEMP_MAX					-0.001 (-0.58)	-0.000 (-0.21)	-0.001 (-0.69)	-0.000 (-0.43)	-0.000 (-0.38)
L5.TEMP_MAX						-0.002+ (-1.71)	-0.001 (-1.54)	-0.001 (-1.42)	-0.001 (-1.60)
L6.TEMP_MAX							-0.000 (-0.11)	0.000 (0.42)	0.000 (0.20)
L7.TEMP_MAX								-0.001 (-0.72)	-0.001 (-0.53)
L8.TEMP_MAX									0.001 (0.64)
L0.TEMP_MIN	0.000 (0.34)	0.000 (0.21)	0.000 (0.25)	0.000 (0.11)	0.000 (0.08)	-0.000 (-0.08)	0.000 (0.14)	0.000 (0.42)	-0.000 (-0.16)
L1.TEMP_MIN		0.000 (0.24)	-0.000 (-0.12)	-0.000 (-0.24)	-0.000 (-0.26)	0.000 (0.01)	-0.000 (-0.44)	-0.001 (-0.79)	-0.001 (-0.72)
L2.TEMP_MIN			0.005*** (4.31)	0.005*** (4.21)	0.005*** (4.20)	0.005*** (3.75)	0.004** (3.20)	0.004*** (3.49)	0.004*** (3.41)
L3.TEMP_MIN				0.000 (0.28)	0.000 (0.29)	0.000 (0.33)	0.001 (0.99)	0.001 (1.07)	0.002 (1.28)
L4.TEMP_MIN					0.001 (1.43)	0.001 (1.15)	0.001 (0.72)	0.000 (0.38)	0.000 (0.28)
L5.TEMP_MIN						0.003* (2.27)	0.002* (2.42)	0.002* (2.09)	0.002+ (1.72)
L6.TEMP_MIN							0.001 (0.53)	0.000 (0.31)	-0.000 (-0.10)
L7.TEMP_MIN								0.001 (1.21)	0.001 (0.67)
L8.TEMP_MIN									0.001 (1.42)
Constant	0.240*** (3.65)	0.192+ (1.93)	0.234+ (1.90)	0.248+ (1.66)	0.282+ (1.67)	0.328* (2.04)	0.196 (1.13)	0.233 (1.43)	0.230 (1.39)
Number of Observations	6,588	6,588	6,588	6,586	6,568	6,524	6,468	6,381	6,223
Number of States	152	152	152	152	152	152	152	152	152
Adjusted R-squared	0.118	0.119	0.124	0.128	0.132	0.137	0.141	0.143	0.146

Note: +: p < 0.10, *: p < 0.05, **: p < 0.01. t-statistics based on robust standard errors in parentheses. L0.UVI stands for the effect of UVI at time t on the cumulative number of COVID-19 deaths at the same time, whereas L1.UVI, L2.UVI and L3.UVI (etc.) stand for the effect of UVI lagged by one, two, or three (etc.) weeks respectively. The same is true for the other variables.

Table S8: Number of observations (Obs.) of countries used in analysis

Country	Obs.	Country	Obs.	Country	Obs.	Country	Obs.
Afghanistan	47	Cuba	39	Kazakhstan	38	Qatar	41
Albania	42	Cyprus	42	Kenya	38	Romania	47
Algeria	55	Czechia	47	Korea, South	73	Russia	50
Andorra	47	Denmark	53	Kosovo	25	San Marino	53
Angola	31	Djibouti	28	Kuwait	34	Sao Tome and Principe	7
Antigua and Barbuda	31	Dominican Republic	50	Kyrgyzstan	33	Saudi Arabia	45
Argentina	48	Ecuador	50	Latvia	35	Senegal	37
Armenia	43	Egypt	61	Lebanon	59	Serbia	45
Australia	68	El Salvador	32	Liberia	34	Sierra Leone	15
Austria	55	Equatorial Guinea	16	Libya	27	Singapore	48
Azerbaijan	50	Estonia	44	Liechtenstein	34	Slovakia	37
Bahamas	35	Eswatini	22	Lithuania	48	Slovenia	46
Bahrain	53	Ethiopia	33	Luxembourg	51	Somalia	30
Bangladesh	43	Finland	48	Malawi	18	South Africa	42
Barbados	33	France	73	Malaysia	52	Spain	66
Belarus	38	Gabon	37	Maldives	9	Sri Lanka	41
Belgium	58	Georgia	34	Mali	26	Sudan	38
Benin	32	Germany	60	Malta	30	Sweden	58
Bolivia	40	Ghana	37	Mauritius	33	Switzerland	55
Bosnia and Herzegovina	46	Greece	54	Mexico	50	Syria	29
Botswana	21	Guatemala	37	Moldova	43	Taiwan*	73
Brazil	52	Guinea	23	Monaco	40	Tanzania	35
Brunei	41	Guinea-Bissau	12	Montenegro	34	Thailand	68
Bulgaria	43	Guyana	39	Morocco	49	Togo	42
Burkina Faso	41	Haiti	31	Netherlands	53	Trinidad and Tobago	37
Burma	24	Honduras	40	New Zealand	40	Tunisia	47
Cabo Verde	31	Hungary	47	Niger	31	Turkey	40
Cameroon	44	Iceland	50	Nigeria	46	US	69
Canada	60	India	58	North Macedonia	47	Ukraine	48
Chad	10	Indonesia	49	Norway	54	United Arab Emirates	49
Chile	47	Iran	61	Oman	38	United Kingdom	63
China	73	Iraq	56	Pakistan	50	Uruguay	37
Colombia	45	Ireland	51	Panama	41	Uzbekistan	36
Congo (Brazzaville)	36	Israel	48	Paraguay	43	Venezuela	37
Congo (Kinshasa)	40	Italy	73	Peru	45	West Bank and Gaza	43
Costa Rica	45	Jamaica	40	Philippines	73	Yemen	8
Cote d'Ivoire	40	Japan	73	Poland	47	Zambia	33
Croatia	50	Jordan	42	Portugal	49	Zimbabwe	31
Total number of observations				6,524			

Table S9: Robustness checks for governmental measures

	Model 10	Model 11
Dependent Variable	COVID-19 Deaths	COVID-19 Deaths
L0.UVI	-0.003+ (-1.83)	-0.001 (-0.45)
L1.UVI	0.000 (0.15)	0.001 (0.53)
L2.UVI	-0.001 (-0.82)	-0.000 (-0.14)
L3.UVI	-0.003* (-2.04)	-0.002 (-1.56)
L4.UVI	-0.003* (-2.08)	-0.004+ (-1.89)
L5.UVI	-0.002 (-1.01)	-0.002 (-0.93)
Long-Run Coefficient	-0.012*** (F: 13.05)	-0.007* (F: 4.24)

Control variables

Time Trend of Growth Rate	Linear	Linear, square and exponential (country-specific)
Country Fixed-Effects	Yes	Yes
Precipitation index	Yes	Yes
Cloud index	Yes	Yes
Ozone level	Yes	Yes
Visibility level	Yes	Yes
Humidity level	Yes	Yes
Temperature (min and max)	Yes	Yes
Governmental Measures	Yes	Yes
Number of Estimates	97 (+136 FE)	96 (+136 FE +408 TSCE)
Number of Observations	6,044	6,044
Number of Countries	136	136
R-squared Within	18.88%	27.23%

Note: +: $p < 0.10$, *: $p < 0.05$, **: $p < 0.01$. t-statistics based on robust standard errors in parentheses. F-statistic for long-run coefficient in parentheses. L0.UVI stands for the effect of UVI at time t on the cumulated number of COVID-19 deaths at the same time, whereas L1.UVI, L2.UVI, L3.UVI, L4.UVI and L5.UVI stand for the effect of UVI lagged by one, two, or three, four and five weeks respectively. FE stands for country fixed-effects, TCSE stands for time country-specific effects.

Table S10: Robustness checks for governmental measures

	Model 12	Model 13
Dependent Variable	CFR	CFR
L0.UVI	-0.002 (-1.11)	-0.001 (-0.32)
L1.UVI	-0.000 (-0.07)	0.001 (0.50)
L2.UVI	-0.003+ (-1.79)	-0.001 (-0.99)
L3.UVI	-0.003+ (-1.87)	-0.002 (-1.33)
L4.UVI	-0.004* (-2.09)	-0.004+ (-1.94)
L5.UVI	-0.001 (-0.46)	-0.001 (-0.41)
Long-Run Coefficient	-0.012** (F: 10.88)	-0.008* (F: 4.00)

Control variables

Time Trend of Growth Rate	Linear	Linear, square and exponential (country-specific)
Country Fixed Effects	Yes	Yes
Precipitation index	Yes	Yes
Cloud index	Yes	Yes
Ozone level	Yes	Yes
Visibility level	Yes	Yes
Humidity level	Yes	Yes
Temperature (min and max)	Yes	Yes
Governmental Measures	Yes	Yes
Number of Estimates	97 (+136 FE)	96 (+136 FE +408 TSCE)
Number of Observations	6,044	6,044
Number of Countries	136	136
R-squared Within	4.73%	13.62%

Note: +: p < 0.10, *: p < 0.05, **: p < 0.01. t-statistics based on robust standard errors in parentheses. F-statistic for long-run coefficient in parentheses. L0.UVI stands for the effect of UVI at time t on the cumulated number of COVID-19 deaths at the same time, whereas L1.UVI, L2.UVI, L3.UVI, L4.UVI and L5.UVI stand for the effect of UVI lagged by one, two, or three, four and five weeks respectively. FE stands for country fixed-effects, TCSE stands for time country-specific effects.

Table S11: Latitude (Lat.) and longitude (Long.) of countries used in analysis

Country	Lat.	Long.	Country	Lat.	Long.	Country	Lat.	Long.
Afghanistan	33.00	65.00	Finland	64.00	26.00	Niger	17.61	8.08
Albania	41.15	20.17	France	8.31	5.43	Nigeria	9.08	8.68
Algeria	28.03	1.66	Gabon	-0.80	11.61	North Macedonia	41.61	21.75
Andorra	42.51	1.52	Georgia	42.32	43.36	Norway	60.47	8.47
Angola	-11.20	17.87	Germany	51.00	9.00	Oman	21.00	57.00
Antigua and Barbuda	17.06	-61.80	Ghana	7.95	-1.02	Pakistan	30.38	69.35
Argentina	-38.42	-63.62	Greece	39.07	21.82	Panama	8.54	-80.78
Armenia	40.07	45.04	Guatemala	15.78	-90.23	Paraguay	-23.44	-58.44
Australia	-32.00	141.23	Guinea	9.95	-9.70	Peru	-9.19	-75.02
Austria	47.52	14.55	Guinea-Bissau	11.80	-15.18	Philippines	13.00	122.00
Azerbaijan	40.14	47.58	Guyana	5.00	-58.75	Poland	51.92	19.15
Bahamas	25.03	-77.40	Haiti	18.97	-72.29	Portugal	39.40	-8.22
Bahrain	26.03	50.55	Honduras	15.20	-86.24	Qatar	25.35	51.18
Bangladesh	23.69	90.36	Hungary	47.16	19.50	Romania	45.94	24.97
Barbados	13.19	-59.54	Iceland	64.96	-19.02	Russia	60.00	90.00
Belarus	53.71	27.95	India	21.00	78.00	San Marino	43.94	12.46
Belgium	50.83	4.00	Indonesia	-0.79	113.92	Sao Tome and Principe	0.19	6.61
Benin	9.31	2.32	Iran	32.00	53.00	Saudi Arabia	24.00	45.00
Bolivia	-16.29	-63.59	Iraq	33.00	44.00	Senegal	14.50	-14.45
Bosnia and Herzegovina	43.92	17.68	Ireland	53.14	-7.69	Serbia	44.02	21.01
Botswana	-22.33	24.68	Israel	31.00	35.00	Sierra Leone	8.46	-11.78
Brazil	-14.24	-51.93	Italy	43.00	12.00	Singapore	1.28	103.83
Brunei	4.54	114.73	Jamaica	18.11	-77.30	Slovakia	48.67	19.70
Bulgaria	42.73	25.49	Japan	36.00	138.00	Slovenia	46.15	15.00
Burkina Faso	12.24	-1.56	Jordan	31.24	36.51	Somalia	5.15	46.20
Burma	21.92	95.96	Kazakhstan	48.02	66.92	South Africa	-30.56	22.94
Cabo Verde	16.54	-23.04	Kenya	-0.02	37.91	Spain	40.00	-4.00
Cameroon	3.85	11.50	Korea, South	36.00	128.00	Sri Lanka	7.00	81.00
Canada	51.60	-93.35	Kosovo	42.60	20.90	Sudan	12.86	30.22
Chad	15.45	18.73	Kuwait	29.50	47.75	Sweden	63.00	16.00
Chile	-35.68	-71.54	Kyrgyzstan	41.20	74.77	Switzerland	46.82	8.23
China	32.83	111.65	Latvia	56.88	24.60	Syria	34.80	39.00
Colombia	4.57	-74.30	Lebanon	33.85	35.86	Taiwan*	23.70	121.00
Congo (Brazzaville)	-4.04	21.76	Liberia	6.43	-9.43	Tanzania	-6.37	34.89
Congo (Kinshasa)	-4.04	21.76	Libya	26.34	17.23	Thailand	15.00	101.00
Costa Rica	9.75	-83.75	Liechtenstein	47.14	9.55	Togo	8.62	0.82
Cote d'Ivoire	7.54	-5.55	Lithuania	55.17	23.88	Trinidad and Tobago	10.69	-61.22
Croatia	45.10	15.20	Luxembourg	49.82	6.13	Tunisia	34.00	9.00
Cuba	22.00	-80.00	Malawi	-13.25	34.30	Turkey	38.96	35.24
Cyprus	35.13	33.43	Malaysia	2.50	112.50	US	37.09	-95.71
Czechia	49.82	15.47	Maldives	3.20	73.22	Ukraine	48.38	31.17
Denmark	63.29	-13.34	Mali	17.57	-4.00	United Arab Emirates	24.00	54.00
Djibouti	11.83	42.59	Malta	35.94	14.38	United Kingdom	24.55	-43.90
Dominican Republic	18.74	-70.16	Mauritius	-20.20	57.50	Uruguay	-32.52	-55.77
Ecuador	-1.83	-78.18	Mexico	23.63	-102.55	Uzbekistan	41.38	64.59
Egypt	26.00	30.00	Moldova	47.41	28.37	Venezuela	6.42	-66.59
El Salvador	13.79	-88.90	Monaco	43.73	7.42	West Bank and Gaza	31.95	35.23
Equatorial Guinea	1.50	10.00	Montenegro	42.50	19.30	Yemen	15.55	48.52
Estonia	58.60	25.01	Morocco	31.79	-7.09	Zambia	-15.42	28.28
Eswatini	-26.52	31.47	Netherlands	21.41	-53.01	Zimbabwe	-20.00	30.00
Ethiopia	9.15	40.49	New Zealand	-40.90	174.89			

10 References

1. Rubin, D. B. Causal inference using potential outcomes: Design, modeling, decisions. *J. Am. Stat. Assoc.* **100**, 322–331 (2005).
2. Conticini, E., Frediani, B. & Caro, D. Can atmospheric pollution be considered a co-factor in extremely high level of SARS-CoV-2 lethality in Northern Italy? *Environ. Pollut.* **261**, 114465 (2020).
3. Hale, T., Petherick, A., Phillips, T. & Webster, S. Variation in government responses to COVID-19. *Blavatnik Sch. Gov. Work. Pap.* **31**, (2020).
